# Using Digital PCR to Unravel the Occurrence of Piroplasmids, *Bartonella* spp., and *Borrelia* spp. in Wild Animals from Brazil

**DOI:** 10.3390/pathogens14060567

**Published:** 2025-06-06

**Authors:** Ana Cláudia Calchi, Anna Claudia Baumel Mongruel, Fernanda Beatriz Pereira Cavalcanti, Lilliane Bartone, José Maurício Barbanti Duarte, Emília Patrícia Medici, Danilo Kluyber, Mayara G. Caiaffa, Mario Henrique Alves, Arnaud Leonard Jean Desbiez, Taciana Fernandes Souza Barbosa Coelho, Rosangela Zacarias Machado, Edward B. Breitschwerdt, Ricardo G. Maggi, Marcos Rogério André

**Affiliations:** 1 Vector-Borne Bioagents Laboratory (VBBL), Department of Pathology, Reproduction and One Health, School of Agricultural and Veterinarian Sciences (FCAV), São Paulo State University (UNESP), Jaboticabal 14884-900, SP, Brazil; ana.calchi@unesp.br (A.C.C.);; 2 Programa de Pós-Graduação em Ciências Veterinárias, FCAV-UNESP, São Paulo State University (UNESP), Jaboticabal 14884-900, SP, Brazil; 3 Intracellular Pathogens Research Laboratory, Comparative Medicine Institute, College of Veterinary Medicine, North Carolina State University, Raleigh, NC 27607, USA; 4 Deer Research and Conservation Center (NUPECCE), São Paulo State University (UNESP), Jaboticabal 14884-900, SP, Brazil; 5 Lowland Tapir Conservation Initiative (LTCI), Instituto de Pesquisas Ecológicas (IPÊ), Nazaré Paulista 12960-000, SP, Brazil; 6 IUCN SSC Tapir Specialist Group (TSG), Campo Grande 79046-150, MS, Brazil; 7 ICAS—Instituto de Conservacao de Animais Silvestres, Campo Grande, Mato Grosso do Sul 79040-290, MS, Brazil; 8 Naples Zoo at the Caribbean Gardens, Naples, FL 34102, USA; 9 São Paulo Institute of Tropical Medicine, Faculdade de Medicina, University of São Paulo, São Paulo 05403-000, SP, Brazil; 10 Research and Development Institute, Maladies Infectieuses et Vecteurs: Ecologie, Génétique, Evolution et Controlê, Montpellier University, F-34394 Montpellier, France; 11 Department of Veterinary Medicine, University of Bari, 70121 Bari, Italy; 12 RZSS—The Royal Zoological Society of Scotland, Murrayfield, Edinburgh EH12, UK; 13 Section of Arbovirology and Hemorrhagic Fevers, Coordinator of the Rabies Diagnosis Laboratory, Evandro Chagas Institute MS-SVS, São Brás, Belém 66093-020, PA, Brazil

**Keywords:** PCR techniques, dPCR, hemoparasites, vector-borne agents, wild animals

## Abstract

Piroplasmids (*Babesia* spp., *Rangelia* spp., *Theileria* spp., *Cytauxzoon* spp.) are tick-borne apicomplexan protozoa that infect, depending on the species, erythrocytes and leucocytes in a wide variety of mammals and birds. The genera *Bartonella* and *Borrelia* include vector-borne bacteria that can infect and cause disease in both animals and humans. Detection of hemotropic bacteria and piroplasmids in wild animals is often challenging due to low bacteremia or parasitemia. Digital (d)PCR has proven to be an effective modality for the detection and quantification of DNA of hemotropic pathogens with low parasitemia. This study compared dPCR results from 366 biological samples from seven different Brazilian wild animal groups (5 Xenarthra species, 5 deer species, 3 felid species, 1 canid species, 3 rodent species, 1 bat species, 1 tapir species, and 12 bird species) to two other molecular diagnostic techniques: quantitative real-time (qPCR) and nested (nPCR). For this study, DNA extracted from wild animal blood and spleen samples were subjected to a multiplex dPCR assay for piroplasmids, *Bartonella* spp., and *Borrelia* spp. For comparison, the same primers and probes for each agent were used in qPCR assays. Additionally, an nPCR based on the 18S rRNA gene for piroplasmids was performed. The proportions of positive results obtained using dPCR were 85.5% for piroplasmids, 33.6% for *Bartonella* spp., and 16.7% for *Borrelia* spp. For all tested agents, dPCR proved to be the technique with the highest sensitivity, making it a useful tool for screening vector-borne agents in biological samples from wild animals with low parasitemia.

## 1. Introduction

Arthropod-borne diseases currently account for 22.8% of emerging diseases [1]. Climate, environment, and ecology changes can disrupt the natural cycles of microorganisms, leading to the emergence of new diseases [2,3]. These changes also broaden the distribution of vectors and increase pathogen proximity among wildlife, domestic animals, and humans. As a result, closer interactions among reservoirs, vectors, and susceptible hosts are expected, facilitating the spread of diseases and presenting significant challenges to public and animal health [2,3].

*Bartonella* spp. (Hyphomicrobiales: Bartonellaceae) are Gram-negative fastidious facultative intracellular alphaproteobacteria that are primarily transmitted by arthropods (e.g., fleas, lice, ticks, and sand flies), as well as through scratches and bites [4,5,6]. *Borrelia* spp. (Spirochaetales: Spirochaetaceae) are spirochete bacteria transmitted by ixodid and argasid ticks and lice. Three distinct groups of *Borrelia* spp. are currently recognized: the Lyme group, the relapsing fever group, and the Echidna-Reptile group [7,8,9]. Piroplasmids (Order Piroplasmida) are apicomplexan protozoa transmitted by ixodid ticks. This group consists of two families: Babesiidae (genera *Babesia* and *Rangelia*) and Theileriidae (genera *Theileria* and *Cytauxzoon*) [10,11]. These three pathogen groups contain zoonotic species that can cause disease in domestic and wild animals and human patients [7,8,12,13].

Brazil is a country with a highly diverse fauna [14]. Research into these agents among wild animals has increased substantially in recent years, resulting in the discovery of putative new species [15,16,17,18,19,20,21,22,23,24,25,26,27,28,29,30,31,32,33,34,35,36]. Despite numerous published studies, molecular detection of pathogen DNA in wild animals remains a diagnostic challenge due to the low parasitemia associated with these agents, which underestimates organism prevalence in epidemiological and clinical studies [3,11,19].

Currently, techniques known as third-generation PCR are being developed to increase the sensitivity of molecular assays. The most notable of these, digital PCR (dPCR), shares the same amplification principle as real-time PCR (qPCR) by using hydrolysis probes, but it allows samples to be fractionated into micro-well plates, capillaries (dPCR), or emulsions (digital droplet (dd)PCR) [37,38]. Fractionation enables separate amplification reactions in each partition, enhancing the detection of low concentrations of DNA, such as in samples from animals with low parasitemia or in the detection of rare genetic mutations [39,40,41]. Additionally, absolute quantification is achieved without the need for a standard curve using Poisson statistics to compare the number of positive and negative partitions [42,43,44,45]. Another advantage of this technique is its effectiveness in diluting out potential PCR inhibitors compared to qPCR [38,45,46,47,48]. The main disadvantages are its high cost and the inability to sequence dPCR-positive samples [39,40,41,42].

To date, dPCR has been used to enhance the molecular detection of different hemoparasites such as *Borrelia* spp. [49,50,51], *Bartonella* spp. [51,52], *Anaplasma phagocytophilum* [53], *Coxiella burnetii* [54], *Ehrlichia canis* [40], *Trypanosoma cruzi* [55], *Leishmania* spp. [56,57], *Plasmodium* spp. [58,59,60], and piroplasmids [51,61,62,63]. The use of this technique is still not widespread worldwide, including in Brazil. Due to the enhanced sensitivity, this study aimed to compare the positivity rates obtained using different PCR techniques (nested PCR–nPCR, qPCR, and dPCR) for piroplasmids, *Bartonella* spp., and *Borrelia* spp. in order to determine which technique is most suitable for detecting the selected vector-borne agents in wild animal blood and spleen samples.

## 2. Materials and Methods

### 2.1. Samples Analyzed

In total, 366 blood (*n* = 116), buffy coat (*n* = 174), and spleen (*n* = 76) DNA samples from wild animals in Brazil were analyzed. The animal species included: 174 deer (139 *Blastocerus dichotomus*, 6 *Ozotocerus bezoarticus*, 22 *Subulo gouazoubira*, 3 *Mazama americana*, and 4 *Mazama bororo*), 51 vampire bats (*Desmodus rotundus*), 11 *Cerdocyon thous*, 35 felids (12 *Leopardus pardalis*, 21 *Panthera onca*, and 2 *Puma concolor*), 44 Xenarthrans (17 *Myrmecophaga tridactyla*, 9 *Tamandua tetradactyla*, 7 *Dasypus novemcinctus*, 5 *Euphractus sexcinctus*, and 6 *Priodontes maximus*), 24 tapirs (*Tapirus terrestris*), 9 rodents (4 *Oecomys mamorae*, 4 *Thrichomys fosteri*, and 1 *Clyomis laticeps*), and 18 birds (1 *Pseudoseisura unirufa*, 3 *Saltator coerulescens*, 2 *Turdus leucomelas*, 1 *Cercomacra melanaria*, 2 *Ramphocelus carbo*, 1 *Basileuterus flaveolus*, 3 *Leptotila verreauxi*, 1 *Icterus cayanensis*, 1 *Lepidocolaptes angustirostris*, 1 *Fumarius rufus*, 1 *Fumarius leucopus*, and 1 *Cyamocorax chrysops*). All of the samples had been used in previously published studies [23,25,27,28,31,32,33,35].

### 2.2. Molecular Assays

#### 2.2.1. dPCR Assay (Multiplex for *Piroplasmids*, *Bartonella* spp., *Borrelia* spp., and Housekeeping Gene)

A multiplex dPCR assay for piroplasmids, *Bartonella* spp., *Borrelia* spp., and the housekeeping gene was performed as previously described, with modifications [51]. All primer and probe sequences are described in Table 1.

The assays were performed using a QIAcuity Nanoplate 8.5k 96-well format (QIAGEN, Germantown, MD, USA). Each reaction (12 μL final volume) included 4.5 μL of molecular-grade water, 3 μL of 4X QIAcuity Probe PCR Master Mix (QIAGEN), 0.15 μL of each species-specific primer at 100 μM (IDT-DNA Technologies, Coralville, IA, USA), 0.05 μL of each species-specific probe at 100 μM, and 5 μL of DNA sample. Molecular-grade water and DNA from a negative dog blood sample were used as negative controls, while *Babesia microti*, *Bartonella henselae* SA1, and *Borrelia burgdorferi* B31 DNA were used as positive controls.

Amplification was performed on a QIAcuity One 5plex Platform System using 8.5k 96-well nanoplates under the following conditions: 95 °C for 2 min, followed by 40 cycles of denaturation at 95 °C for 20 s, and annealing/extension at 64 °C for 20 s. The detection of each species was assessed by reading the fluorescent signals (dots) using the following color codes: FAM (green) for *Bartonella* spp., HEX (yellow) for *Borrelia* spp., TAMRA (orange) for the housekeeping gene, and Cy5 (crimson) for piroplasmids. The fluorescent signal threshold was assessed as positive or negative for DNA amplification. The results were analyzed using QIAcuity Software Suite version 2.2.0.26. Partitions with fluorescence signals below the defined threshold were classified as negative, while those above the threshold were considered positive. Samples were deemed positive when they contained one or more positive partitions, provided the signal was clearly above the threshold, in order to minimize the risk of false positive results. The number of copies per microliter (copies/µL) was calculated by the software based on the number of positive and negative partitions using Poisson statistics.

#### 2.2.2. qPCR Assay (Multiplex for Piroplasmids, *Bartonella* spp., and *Borrelia* spp.)

Real-time qPCR assays were performed using the same primers and probes described above for the detection of piroplasmids, *Bartonella* spp., and *Borrelia* spp. in order to compare the results obtained using both PCR techniques.

Multiplex real-time qPCR was performed using a reaction mixture with a final volume of 25 μL per reaction containing: 0.25 μL of each primer for *Bartonella* spp. and *Borrelia* spp., 0.375 μL of each primer for piroplasmids at 100 μM (IDT-DNA Technologies, Coralville, IA, USA), 0.125 μL of 100 μM of each species-specific probe, 12.5 μL of 2X SsoAdvanced Probe Master Mix (Bio-Rad, Hercules, CA, USA), 5 μL of template DNA, and 5.875 μL of molecular-grade water (QIAGEN, Germantown, MD, USA). Molecular-grade water and DNA from a negative dog blood sample were used as PCR negative controls. *Babesia microti*, *Bartonella henselae* SA1, and *Borrelia burgdorferi* B31 DNA were used as positive controls.

Amplification was performed using a Bio-Rad CFX 96-well Opus PCR system under the following conditions: 95 °C for 3 min, followed by 45 cycles of denaturation at 94 °C for 10 s, annealing at 68 °C for 10 s, and extension at 72 °C for 10 s. Fluorescent signals were used to assess the detection of each species, using FAM (green) for *Bartonella* spp., HEX (yellow) for *Borrelia* spp., and Cy5 (crimson) for piroplasmids. The fluorescent signal threshold value (Cq) was recorded to determine positive and negative DNA amplification.

Additionally, positive samples were purified and sequenced by Sanger’s method (Genewiz, Azenta, Research Triangle Park, NC, USA) and analyzed using SnapGene Version 7.0.2.

#### 2.2.3. Nested (n)PCR Assays for Piroplasmid Detection

nPCR was performed to amplify approximately an 800 bp fragment of the 18S rRNA gene of piroplasmids using the primers BTF1 (5′-GGCTCATTACAACAGTTATAG-3′) and BTR1 (5′-CCCAAAGACTTTGATTTCTCTC-3′) in the first reaction, and BTF2 (5′-CCGTGCTAATTGTAGGGCTAATAC-3′) and BTR2 (5′-GGACTACGACGGTATCTGATCG-3′) in the second reaction, as described by Jefferies et al. (2007) [64].

The amplification reaction was carried out with a total final volume of 25 μL containing the following components: 5 μL of sample DNA, 0.2 mM of each deoxynucleotide, 0.5 μM of each primer oligonucleotide, 1.5 mM of magnesium chloride, 0.75 U of Taq DNA polymerase, PCR buffer, and sterile ultrapure water to complete the volume. For the second amplification, 1 μL of the product from the first reaction was used.

The thermal cycling conditions for the first reaction were the following: initial denaturation at 94 °C for 3 min, followed by 45 cycles of 94 °C for 30 s, 58 °C for 20 s, and 72 °C for 30 s, with a final extension at 72 °C for 7 min. For the second reaction, the same thermal conditions were used, with the exception that the annealing temperature was set to 62 °C. Ultrapure sterile water was used as a negative control in all PCR assays, while *Babesia vogeli* DNA (Jaboticabal sample) was used as a positive control.

### 2.3. Statistical Analysis

To compare the performance of the assays for detecting piroplasmids, Fleiss’ Kappa test was initially used to assess the level of agreement between all tests (dPCR, qPCR, and nPCR), beyond what would be expected by chance [65]. As a post hoc analysis, agreement beyond chance between test pairs was evaluated using Cohen’s Kappa test [66]. For the other agents (*Bartonella* spp. and *Borrelia* spp.), Cohen’s Kappa test was applied.

The interpretation of Kappa (κ) values was based on the criteria outlined by McHugh (2012) [67], which are as follows: values below zero indicate agreement below that expected by chance (disagreement); values between 0.00 and 0.20 suggest no agreement; values between 0.21 and 0.39 represent minimal agreement; values between 0.40 and 0.59 indicate weak agreement; values between 0.60 and 0.79 reflect moderate agreement; values between 0.80 and 0.90 indicate strong agreement; and values above 0.90 reflect almost perfect agreement.

To assess the difference between the proportions obtained by the tests, McNemar’s test [68] was performed for each pair of tests. All statistical analyses were carried out using RStudio 4.2.3 software with the *psych* [69] and *irr* [70] packages.

The sensitivity and specificity of the assays evaluated (qPCR and nPCR for piroplasmids; qPCR for *Bartonella* spp. and *Borrelia* spp.) were determined using dPCR as the gold standard due to its superior sensitivity compared to the other assays [51]. Finally, the Matthews correlation coefficient (MCC) [71] was calculated for each pair, using dPCR as the gold standard, to assess the performance of the assays. The MCC values range from −1 to 1, with extreme values indicating maximum performance (1) or total failure in classification (−1). A value of zero indicates performance equivalent to random choice [71].

**Figure 1:**
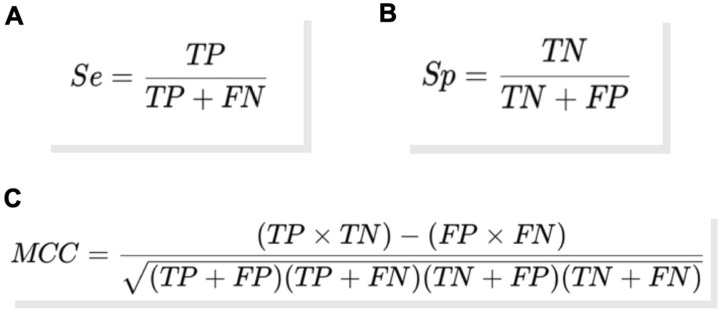
Formulas used to calculate sensitivity (**A**), specificity (**B**), and Matthews correlation coefficient (**C**). In these formulas, TP represents the number of true positive samples (identified as positive by the gold standard), FN represents the number of false negative samples (identified as negative by the assay evaluated but as positive by the gold standard), TN represents the number of true negative samples (identified as negative by the gold standard), and FP represents the number of false positive samples (identified as positive by the assay evaluated but as negative by the gold standard).

The formulas shown in Figure 1 were used to calculate sensitivity (Se), specificity (Sp), and MCC.

## 3. Results

### 3.1. Positivity for Each Technique

Table 2 depicts the number of positive animals for each group and species based on the agent analyzed (piroplasmids, *Bartonella* spp., and *Borrelia* spp.). For all agents, dPCR had a higher number of positive samples compared to the other techniques, with the exception of piroplasmid detection in Xenarthra species, where nPCR detected one more positive sample (*n* = 22) than dPCR (*n* = 21).

**Figure 2:**
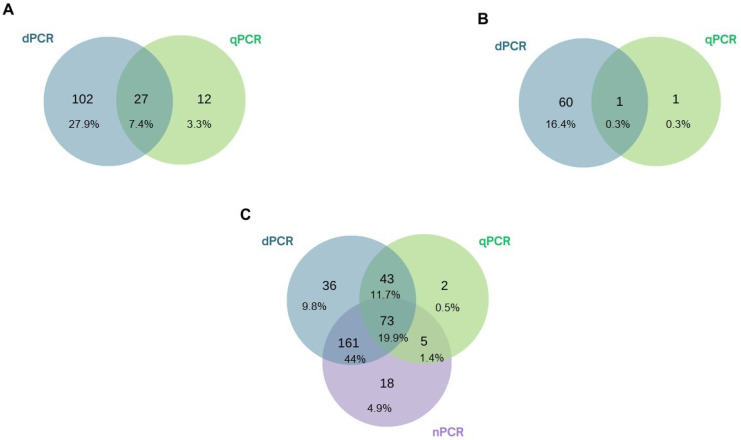
Venn diagram illustrating the samples positive for *Bartonella* spp. (**A**), *Borrelia* spp. (**B**), and piroplasmids (**C**), as identified by the tests analyzed in this study.

To better understand the positivity rates and compare the number of positive samples across techniques, Venn diagrams were generated (Figure 2). These diagrams showed that the number of positive samples was higher when using the dPCR technique for all target agents. For *Bartonella* spp. (Figure 2A) and *Borrelia* spp. (Figure 2B), only 27 and 1 samples, respectively, were positive when both techniques were used (dPCR and qPCR). Additionally, 12 and 1 samples were exclusively positive when qPCR was used for *Bartonella* spp. and *Borrelia* spp., respectively.

In the case of piroplasmids (Figure 2C), 73 samples tested positive across all three techniques performed (dPCR, nPCR, and qPCR). A larger number of samples (*n* = 161) was positive when both dPCR and nPCR were used, while 43 samples were positive when both dPCR and qPCR were used. Only 25 samples were positive using techniques other than dPCR—18 in nPCR alone, 2 in qPCR alone, and 5 in both nPCR and qPCR.

Given that the objective of this study was to compare the positivity between techniques, and considering the absence of standard calibration curves for qPCR, absolute quantification of the samples using this method was not performed. Nonetheless, the number of positive partitions, the concentration expressed as copies per microliter (copies/µL) of the target fragment obtained via dPCR, and the quantification cycle (Cq) values derived from qPCR were duly documented.

**Figure 3:**
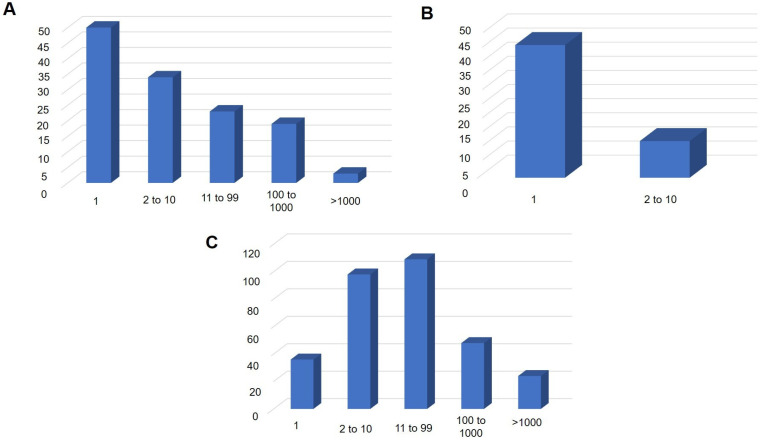
Number of samples positive for *Bartonella* spp. (**A**), *Borrelia* spp. (**B**), and piroplasmids (**C**) across different ranges of positive partitions. The x-axis represents the range of the number of positive partitions, while the y-axis shows the number of samples corresponding to each range of positive partitions.

For *Bartonella* spp. and *Borrelia* spp., most samples exhibited only a single positive partition in the dPCR assays (*n* = 50 and *n* = 47, respectively) (Figure 3A,B). For *Bartonella* spp., the lowest concentration of the amplified 16S–23S rRNA gene fragment was 0.356 copies/µL, while the highest concentration reached 4150.8 copies/µL in the dPCR assays (Table S1). In the qPCR assays, the mean Cq value was 30.86, with a minimum of 14.62 and a maximum of 44.7 (Table S1). For *Borrelia* spp., dPCR detected a minimum of 0.356 copies/µL and a maximum of 2.636 copies/µL of the target fragment (Table S2). In the qPCR analyses, Cq values of 43.26 and 44.93 were obtained for the two amplified bat DNA samples (Table S2).

Regarding piroplasmids, the dPCR analyses revealed that the majority of positive samples (*n* = 207) exhibited between 2 and 100 positive partitions, while 20 samples presented with more than 1000 positive partitions (Figure 3C). This technique was capable of detecting as few as 0.356 copies/µL of the 18S rRNA gene fragment, with the highest concentration recorded at 1784.1 copies/µL (Table S3). In the qPCR assays, the mean Cq value was 33.22, ranging from a minimum of 25.12 to a maximum of 43.57 (Table S3).

With regard to the samples that tested positive using qPCR but negative using dPCR, the majority presented Cq values greater than 39. Among the seven piroplasmid samples that were positive only using qPCR, five exhibited Cq values above 39. Similarly, of the twelve *Bartonella* spp. samples that were qPCR-positive only, eight had Cq values exceeding 39. For the two *Borrelia* spp. samples derived from bat DNA that tested positive exclusively using qPCR, both exhibited Cq values above 43.

Only samples with Cq values lower than 38 using qPCR were sent for sequencing to confirm that there was targeted amplification of the group of agents surveyed. In total, 94 and 29 samples were submitted for DNA sequencing for piroplasmids and *Bartonella* spp., respectively. Of these samples, 49 piroplasmid and 12 *Bartonella* spp. were successfully sequenced. The qPCR fragment obtained for piroplasmids, ranging from 50 to 70 bp, had high identity (97 to 100% similarity) with *Theileria* spp. or *Babesia* spp. For *Bartonella* spp., the sequences (approximately 100 bp) were mostly obtained from vampire bats (*Desmodus rotundus*) and were identical to a *Bartonella* spp. sequence deposited from the same host species in Guatemala (MN504749). A sequence obtained from *B. dichotomus* was 98.29% identical to a *Bartonella henselae* sequence found in a flea from Brazil (MT095054). A sequence obtained from *T. fosteri* was identical to a *Bartonella machadoae* sequence (CP087114).

Additional Venn diagrams were generated to compare the results obtained using each technique for the different target agents, considering dPCR-positive samples as those with one or more, two or more, and three or more positive partitions (Supplementary Material Figure S1). For all agents evaluated, the highest concordance between the techniques was observed when samples with one or more positive partitions were considered positive in the dPCR analyses.

### 3.2. Statistical Analyses

#### 3.2.1. Level of Agreement Between Tests

##### Piroplasmids

The Fleiss’ Kappa test indicated that the agreement among the three tests (dPCR vs. qPCR vs. nPCR) was lower than expected by chance; however, this difference was not statistically significant (*p* > 0.05). When analyzing the agreement between pairs of tests, the Cohen’s Kappa test revealed that the levels of agreement were lower than expected by chance, or absent, but all were statistically significant (*p* < 0.05). These results suggested that the agreement levels among assays varied depending upon which pair of tests was being compared. Among all comparisons, the lowest Kappa index was observed between qPCR and nPCR results (k = −0.09), indicating a significant disagreement. The agreement between nPCR and dPCR results was considered minimal (k = 0.20), while the agreement between dPCR and qPCR results was considered absent (k = 0.089). Table 3 summarizes the results from both the Fleiss’ Kappa and Cohen’s Kappa tests.

##### *Bartonella* spp. and *Borrelia* spp.

For the detection of *Bartonella* spp., the Cohen’s Kappa test indicated that the agreement between qPCR and dPCR was absent (k = 0.1887) but statistically significant (*p* = <0.0001; *p* < 0.05). These results indicated a non-random disagreement between these molecular assays, with dPCR presenting superior performance for detecting *Bartonella* spp. compared to qPCR. For *Borrelia* spp., the Cohen’s Kappa test showed that the agreement between qPCR and dPCR was absent (k = 0.0214) and not statistically significant (*p* = 0.4261; *p* > 0.05).

#### 3.2.2. Analysis of Differences Between Proportions

##### Piroplasmids

The difference in proportions between the tests was analyzed in pairs using McNemar’s test. All comparisons yielded statistically significant results (*p* < 0.05), indicating that the PCR assays significantly differed in their ability to detect piroplasmid-positive samples. The most notable difference was observed between dPCR and qPCR (χ² = 117.09), reinforcing a discrepancy in positive results obtained using the two methods. Table 4 summarizes the results of McNemar’s test.

##### *Bartonella* spp. and *Borrelia* spp.

The difference in proportions between qPCR and dPCR, for both *Bartonella* spp. and *Borrelia* spp. detection, was statistically significant (*p* < 0.05), indicating that qPCR and dPCR significantly differed in their ability to amplify the DNA of the targeted organisms in wild animal samples. Table 5 summarizes the results of McNemar’s test for these assays.

#### 3.2.3. Sensitivity and Specificity

##### Piroplasmids

The sensitivity and specificity values for qPCR, compared to the gold standard (dPCR), were 36.82% and 86.27%, respectively. These values also indicated a low capacity to identify positive samples compared to dPCR. For nPCR, the sensitivity and specificity values were 74.60% and 54.90%, respectively, demonstrating an unsatisfactory ability to correctly identify negative results compared to dPCR.

##### *Bartonella* spp. and *Borrelia* spp.

For the *Bartonella* spp. qPCR assay, the sensitivity and specificity values, compared to the gold standard (dPCR), were 20.93% and 94.94%, respectively. These results indicated a poor ability to detect positive samples compared to dPCR, but a high specificity in distinguishing *Bartonella* spp.-negative samples.

For the *Borrelia* spp. detection assay, the sensitivity and specificity values were 1.64% and 99.67%, respectively, indicating an extremely low capacity to detect positive samples when compared to the gold standard, but a high ability to identify negative samples.

#### 3.2.4. Matthews Correlation Coefficient (MCC)

##### Piroplasmids

The MCC was calculated to assess the degree of agreement between the qPCR and nPCR assays compared to the gold standard (dPCR). The value obtained for the comparison between qPCR and dPCR was 0.1693538, while the value for the comparison between nPCR and dPCR was 0.2240352. These values indicated that both qPCR and nPCR had a low correlation with dPCR, although nPCR had a slightly higher correlation.

##### *Bartonella* spp. and *Borrelia* spp.

For the *Bartonella* spp. detection assay, the MCC value for the comparison between qPCR and dPCR was 0.24566722, indicating a low correlation with dPCR. For the *Borrelia* spp. detection assay, the MCC value was −0.0256786, indicating no correlation between the tests.

## 4. Discussion

In this study, we evaluated the performance of dPCR, qPCR, and nPCR in detecting three vector-borne agents in biological samples from wild animals from Brazil, in which low parasitemia was expected. In general, there was no agreement between the molecular tests performed for piroplasmids, *Bartonella* spp., and *Borrelia* spp. nPCR and dPCR had slightly better agreement for the detection of piroplasmids, albeit the level of agreement was still low. The qPCR protocol used demonstrated low sensitivity in detecting the targeted agents investigated in the selected wild animal samples, whereas dPCR proved to be much more sensitive in detecting these selected agents compared to the other two techniques.

Some previous studies contradict our findings, reporting a high level of agreement between results obtained using ddPCR and qPCR [46,57,62]. These studies investigated the detection of *Cryptosporidium* oocysts in fecal samples from animals and humans previously confirmed positive by microscopy [46], *Cytauxzoon* spp. in blood samples from cats with acute cytauxzoonosis [62], and *Leishmania infantum* in spleen samples from dogs exhibiting clinical signs consistent with leishmaniasis [57]. Notably, none of these studies included samples from wild animals, and all analyzed specimens originated from individuals either displaying clinical signs or previously confirmed positive by other diagnostic methods. By contrast, a study published by Lashnits et al. (2021) [72], which compared the sensitivity of *Bartonella* diagnostic assays in dogs with hemangiosarcoma, found that ddPCR was significantly more sensitive than qPCR for the detection of *Bartonella* DNA in blood samples (36% vs. 0%, *p* < 0.0001). Additionally, a study by Belmonte et al. (2016) [73] compared the effectiveness of ddPCR and qPCR in detecting low-abundance mitochondrial DNA (mtDNA) deletions in human samples. The results demonstrated that ddPCR was highly effective and preferable for samples with moderate deletion loads, whereas qPCR was only effective in samples with high deletion levels.

Furthermore, in previous studies targeting helminth DNA in liver samples from small mammals [74] and in sheep fecal samples [75], ddPCR demonstrated superior sensitivity compared to qPCR. For example, the positivity rate for *Echinococcus multilocularis* in small mammal liver samples was 31.13% using ddPCR, versus only 4.72% with qPCR [74]. In another study, the detection rates for *Trichuris* spp. in sheep fecal samples were 80.6% using ddPCR and 72.4% using qPCR [75]. According to the authors, one possible explanation for these differences was the presence of PCR inhibitors in fecal samples, which may interfere with the amplification and detection of parasite DNA using qPCR. However, the relative abundance of the target DNA may also represent an equally important factor influencing detection sensitivity.

In the present study, the discrepancy between the results obtained using dPCR and qPCR, especially in the detection of *Bartonella* spp. and *Borrelia* spp., might be attributed to extremely low parasitemia in the samples, precluding detection of the target DNA using qPCR. Indeed, the low number of positive partitions in dPCR across most samples indicated low parasitemia. It has been demonstrated that ddPCR can quantify smaller amounts of DNA than the standard curves used in qPCR [61]. Additionally, ddPCR has been shown to identify a higher number of positive samples for *Bartonella* spp. and *Candida* spp. compared to qPCR [39,52]. This can be explained by the fact that DNA samples are fractionated and amplification reactions occur individually in each fraction in dPCR, which favors the detection of low copy numbers of the target DNA fragment. By contrast, qPCR measures fluorescent signals, which can sometimes be less sensitive for low-abundance targets [39,40,41,44].

Herein, some samples were positive using qPCR but negative using dPCR for the agents investigated. When analyzed individually, all of these samples had a Cq value above 39, suggesting that the positive qPCR results were likely due to nonspecific amplification.

Regarding the detection of piroplasmids, only 19.9% samples were positive using all three molecular techniques in this study, while 44% samples were only positive using both nPCR and dPCR, and 11.7% were positive using both dPCR and qPCR. The higher number of positive samples observed using nPCR compared to qPCR is noteworthy; however, when comparing the specificities for each test, nPCR yielded a specificity of 54.9%, while qPCR had a specificity of 86.3%. These findings likely indicate that nPCR might have generated false positive results through nonspecific amplification or due to a higher risk of amplicon contamination, as nPCR requires extensive sample processing between reactions.

An enrichment blood culture protocol for the detection of piroplasmids targeting the 18S rRNA gene, applied to DNA extracted from human blood samples and from 7-, 14-, and 21-day enrichment blood cultures, also demonstrated greater sensitivity using dPCR compared to qPCR. However, when qPCR protocols specifically designed for the amplification of species-specific ITS-1 and ITS-2 intergenic regions for *Babesia odocoilei*, *B. microti*, and *B. divergens* were employed, qPCR proved to be highly effective and sensitive for species detection, with the results confirmed by sequencing [76,77].

Most published studies have employed ddPCR rather than dPCR, with the primary distinction being that ddPCR is performed in an oil emulsion, while dPCR relies on micro-well plates or capillary systems [38]. In the present study, dPCR was conducted using the QIAcuity Digital PCR System (QIAGEN), utilizing 8.5k 96-well nanoplates in an effort to reduce costs while evaluating whether the method would offer sufficient sensitivity for detecting hemoparasite DNA in biological samples from wild animals. Although the use of 26k 24-well plates could potentially yield improved results—given that the fourfold increase in partition number may enhance assay sensitivity—the results obtained demonstrated that the chosen approach was both effective and suitable for the study’s objectives.

There remains some disagreement among manufacturers of dPCR and ddPCR platforms regarding the minimum number of positive partitions or droplets required to consider a sample positive. In the present study, samples that had one or more positive partitions were utilized for the three agents studied. Although a low number of positive partitions could reflect false positive testing results, it was likely attributable to the low levels of parasitemia or bacteremia in the tested samples. This conclusion was supported by the low copy numbers of the target fragments obtained for all the agents analyzed using dPCR, with values ranging from as low as 0.356 copies/µL to a maximum of only 4150.8 copies/µL.

The detection of *Bartonella* spp. in deer blood samples was particularly noteworthy, marking it as the first report of this agent in deer from Brazil. Previously, *Bartonella ribC* genotypes closely related to *Bartonella schoenbuchensis* were detected in *Lipoptena mazamae* flies collected from deer in Rio Grande do Sul, Brazil [78]. Another interesting result was the detection of *Borrelia* spp. in the tested animals. Since all of the positive samples showed only one positive partition, this may help to explain why *Borrelia* spp. are rarely reported in wild animals and ectoparasites from Brazil [20,26,79,80]. A new genospecies of *Borrelia burgdorferi*, called ‘*Candidatus* Borrelia paulista’, was found in two cricetid rodents sampled in the state of São Paulo through qPCR and PCR characterization assays using different gene markers (16S rRNA, *flaB*, *ospC*, *clpA*, *nifS*, *pepX*, *pyrG*, *recG*, *rlpB*, and *uvrA*) [26]. Additionally, genotypes of *Borrelia* spp. phylogenetically related to *Borrelia* spp. detected in bats in Colombia were found circulating in five vampire bats sampled in the state of Ceará, Brazil, based on the 16S rRNA and *flaB* genes [80]. Our research group recently reported the occurrence of *Bartonella* spp. and *Borrelia theileri* in the same tapir samples reported in this study [34,81]. In these two previous tapir studies, positive rates of 8% and 2% were reported for *Bartonella* spp. and *Borrelia* spp., respectively, using qPCR assays. The present study yielded positive rates of >16% for both genera, reinforcing the greater sensitivity of dPCR in detecting these bacterial agents in wildlife.

High diversity of piroplasmids has been reported in wild animals from Brazil, with five new clades added to the phylogeny of Piroplasmida based on the detection of putative novel species in non-hematophagous bats [22], opossums [21,29], tapirs [25], capybaras [30], and wild rodents [36]. Additionally, previous studies using some of the samples analyzed in the present study have reported the detection of a new *Babesia* species, namely *Babesia pantanalensis*, in *C. thous* blood samples [32], as well as the identification of a new *Theileria* species (*Theileria terrestris*) in tapirs [25]. Moreover, at least two species of *Cytauxzoon*—*Cytauxzoon brasiliensis* and genovariants of *Cytauxzoon felis*—have been reported in ocelots and jaguars [35]. The dPCR protocol employed in this study proved to be both useful and sensitive for detecting the diverse range of piroplasmids circulating in the country, as it yielded positive results across all analyzed animal orders. Furthermore, this protocol detected the presence of piroplasmids in all rodent samples and in some bird samples that had tested negative using nPCR, highlighting its potential as a reliable method for detecting these parasites in wildlife hosts.

## 5. Conclusions

This study demonstrated that dPCR has high sensitivity and is a very useful tool for detecting vector-borne agents in biological samples from wild animals with parasitemia levels that range from very low to very high. Additionally, qPCR was not found to be a sensitive technique for the types of samples examined in this study. nPCR exhibited high sensitivity but moderate specificity for detecting piroplasmid DNA when compared to dPCR.

## Figures and Tables

**Table 1:** Description of primers, probes, and amplicon sizes of multiplex dPCR assays.

Agent	Gene	Size	Primer Sequences	Probe Sequences
*Bartonella* spp.	16S-23S rRNA (ITS)	90–120 bp	BsppITS325s 5′-CCTCAGATGATGATCCCAAGCCTTCTGGCG-3′ BsppITS543as 5′-AATTGGTGGGCCTGGGAGGACTTG-3′	BsppITS500probe 5′-FAM-GTTAGAGCGCGCGCTTGATTAAG-BHQ1-3′
*Borrelia* spp.	16S-23S rRNA (ITS)	104 bp	BobulITS120s 5′-AGGTCATTTTGGGGGTTTAGCTCAGTTGGCT-3′ BobulITS200as 5′-AATGGAGGTTAA GGGACTCGAACCCT-3′	BobulITS160probe 5′-HEX-CGGCTTTGCAAGCCGAGGGTCAAG-BHQ2-3′
Piroplasmids	18S rRNA	125–138 bp	Piro18S-238s 5′-TCGGTGATTCATAATAAACTRGCGAATCGC-3′ Piro18S-380as 5′-AATCGAACCCCAATTCCCCGTTACCCG-3′	Piro18S-340probe 5′ CY5-GACGGTAGGGTATTGGCCTACCG-BHQ2-3′
Housekeeping gene	B-Raf Proto-Oncogene (BRAF) gene	120 bp	CaFeBRAF-15s 5′-TCAYGA AGACCTCACAGTAAAAATAGG T-3′ CaFeBRAF-110as 5′-GATCCAGACAAC TGTTCA AACTGATG-3′	CaFeBRAF-50 5′-TAMRA-GTCTAGCCACAGTGAAATCTCGATG-BHQ2-3′

**Table 2:** Number of animals positive for each agent and test performed.

	Number of Positive Samples						
	*Bartonella* spp.		*Borrelia* spp.		Piroplasmids		
Wild Animal Group	dPCR	qPCR	dPCR	qPCR	nPCR	dPCR	qPCR
Deer (Total)	56 (32.18%)	12 (6.9%)	25 (14.37%)	0	137 (78.73%)	154 (88.51%)	51 (29.31%)
*Subulo gouazoubira*	6	2	6	0	22	19	2
*Mazama rufa*	0	0	1	0	3	2	0
*Mazama jucunda*	2	0	3	0	4	4	0
*Blastocerus dichotomus*	45	10	15	0	102	124	47
*Ozotocerus bezoarticus*	3	0	0	0	6	5	2
*Desmodus rotundus* (Total)	46 (90.2%)	17 (33.33%)	14 (27.45%)	2 (3.92%)	42 (82.35%)	47 (92.15%)	16 (31.37%)
*Cerdocyon thous* (Total)	3 (27.27%)	1 (9.09%)	1 (9.09%)	0	3 (27.27%)	11 (100%)	9 (81.81%)
Felids (Total)	6 (17.14%)	1 (2.86%)	7 (20%)	0	29 (82.86%)	34 (97.14%)	19 (54.29%)
*Leopardus pardalis*	1	0	1	0	9	12	10
*Panthera onca*	4	1	4	0	18	21	9
*Puma concolor*	1	0	2	0	2	2	0
Xenarthra (Total)	6 (13.64%)	6 (13.64%)	6 (13.67%)	0	22 (50%)	21 (47.73%)	14 (31.82%)
*Myrmecophaga tridactyla*	0	2	1	0	12	10	6
*Tamandua tetradactyla*	1	0	1	0	4	3	1
*Euphractus sexcinctus*	3	1	1	0	1	3	2
*Dasypus novemcinctus*	2	2	2	0	4	2	3
*Priodontes maximus*	0	1	1	0	1	3	2
Birds (Total)	4 (22.22%)	1 (5.56%)	2 (11.11%)	0	8 (44.44%)	13 (72.22%)	6 (33.33%)
*Pseudoseisura unirufa*	0	0	0	0	1	1	1
*Saltator coerulescens*	2	0	1	0	2	2	2
*Turdus leucomelas*	1	0	0	0	1	1	0
*Cercomacra melanaria*	0	0	0	0	1	1	0
*Ramphoceluls carbo*	0	0	0	0	1	0	0
*Basileuterus flaveolus*	0	0	0	0	1	1	0
*Leptotila verreauxi*	0	1	0	0	1	2	1
*Icterus cayanensis*	0	0	0	0	0	1	1
*Lepidocolaptes angustirostris*	0	0	1	0	0	1	0
*Fumarius rufus*	0	0	0	0	0	1	0
*Fumarius leucopus*	0	0	0	0	0	1	0
*Cyamocorax chrysops*	1	0	0	0	0	1	1
*Tapirus terrestris* (Total)	4 (16.67%)	0	4 (16.67%)	0	16 (66.67%)	24 (100%)	4 (16.67%)
Rodents (Total)	4 (44.44%)	1 (11.11%)	2 (22.22%)	0	0	9 (100%)	4 (44.44%)
*Oecomys mamorae*	3	0	0	0	0	4	3
*Thrichomys fosteri*	1	1	1	0	0	4	1
*Clyomis laticeps*	0	0	1	0	0	1	0
**Total**	129 (35.26%)	39 (10.66%)	61 (16.67%)	2 (0.55%)	257 (70.22%)	313 (85.52%)	123 (33.61%)

**Table 3:** Results obtained in the Fleiss’ Kappa and Cohen’s Kappa analyses of the agreement between test results for piroplasmids.

Test	Comparison	Kappa (k)	*p*-Value
Fleiss’ Kappa	dPCR vs. qPCR vs. nPCR	−0.0517	0.0865
Cohen’s Kappa	qPCR vs. nPCR	−0.0935	**0.0185**
	nPCR vs. dPCR	0.2010	**<0.0001**
	dPCR vs. qPCR	0.0896	**0.0012**

*p*-value is considered significant when <0.05.

**Table 4:** Analysis of the difference between the proportions obtained in the tests for detecting piroplasmids evaluated in this study.

Comparation	χ^2^	*p*-Value
qPCR vs. nPCR	79.101	**<0.0001**
nPCR vs. qPCR	30.447	**<0.0001**
dPCR vs. qPCR	177.09	**<0.0001**

The *p*-value is considered significant when <0.05; χ² = chi-squared value.

**Table 5:** Analysis of the difference between the proportions obtained in the tests for detecting *Bartonella* spp. and *Borrelia* spp. evaluated in this study.

Agent	Comparation	χ^2^	*p*-Value
*Bartonella* spp.	qPCR vs. dPCR	69.482	**<0.0001**
*Borrelia* spp.	qPCR vs. dPCR	55.148	**<0.0001**

The *p*-value is considered significant when <0.05; χ² = chi-squared value.

## Data Availability

Data are contained within the article and Supplementary Materials.

## Supplementary Materials

The following supporting information can be downloaded at: https://www.mdpi.com/article/10.3390/pathogens14060567/s1, Table S1: Results obtained for each test carried out for *Bartonella* spp.; Table S2: Results obtained for each test carried out for *Borrelia* spp.; Table S3: Results obtained for each test carried out for piroplasmids; Figure S1: Venn diagrams comparing the positivity between different techniques for the detection of *Bartonella* spp. (A), *Borrelia* spp. (B), and piroplasmids (C), considering samples as positive using dPCR when presenting one or more, two or more, or three or more positive partitions in the assays performed.

## Abbreviations

The following abbreviations are used in this manuscript:

dPCR	digital PCR
qPCR	quantitative PCR
nPCR	nested PCR
ddPCR	droplet digital PCR
MCC	Matthews correlation coefficient
