# Supplementary material for: Using Digital PCR to Unravel the Occurrence of Piroplasmids, Bartonella spp., and Borrelia spp. in Wild Animals from Brazil
Source: Pathogens. 2025 Jun 6;14(6):567. doi: 10.3390/pathogens14060567 (PMC12195830; doi:10.3390/pathogens14060567)
Supplement: Supplementary file 1 [file pathogens-14-00567-s001.zip › pathogens-3633750-supplementary.pdf]

Supplementary Material

Table S1: Results obtained for each test carried out for *Bartonella* spp.

		dPCR			qPCR	
**Animal species**	**ID**	**Result**	**Positive partitions**	**Conc. (copies/µL)**	**Result**	**CQ**
Subulo gouazoubira	182M	Negative	0	0	Negative	N/A
Mazama jucunda	215M	Negative	0	0	Negative	N/A
Mazama rufa	270M	Negative	0	0	Negative	N/A
Blastocerus dichotomus	63B	Positive	1246	602.2	Positive	44.53
Blastocerus dichotomus	75B	Positive	7	2.655	Negative	N/A
Blastocerus dichotomus	120B2	Positive	1	0.378	Negative	N/A
Blastocerus dichotomus	121B2	Negative	0	0	Negative	N/A
Blastocerus dichotomus	222B3	Negative	0	0	Negative	N/A
Blastocerus dichotomus	226B3	Negative	0	0	Negative	N/A
Desmodus rotundus	91MO	Positive	36	16.25	Negative	N/A
Desmodus rotundus	8MO	Positive	1	0.375	Negative	N/A
Cerdocyon thous	2L	Negative	0	0	Negative	N/A
Leopardus pardalis	8L	Negative	0	0	Negative	N/A
Leopardus pardalis	22L	Negative	0	0	Negative	N/A
Cerdocyon thous	25L	Negative	0	0	Negative	N/A
Myrmecophaga tridactyla	59T	Negative	0	0	Negative	N/A
Myrmecophaga tridactyla	62T	Negative	0	0	Positive	40.71
Tamandua tetradactyla	71T	Negative	0	0	Negative	N/A
Dasypus novemcinctus	16T	Negative	0	0	Negative	N/A
Priodontes maximus	153T	Negative	0	0	Positive	43.95
Priodontes maximus	159T	Negative	0	0	Negative	N/A
Priodontes maximus	160T	Negative	0	0	Negative	N/A
Ozotocerus bezoarticus	06O	Negative	0	0	Negative	N/A
Ozotocerus bezoarticus	07O	Positive	1	0.387	Negative	N/A
Ozotocerus bezoarticus	08O	Positive	1	0.395	Negative	N/A
Ozotocerus bezoarticus	09O	Negative	0	0	Negative	N/A
Ozotocerus bezoarticus	10O	Positive	1	0.396	Negative	N/A
Subulo gouazoubira	154M	Negative	0	0	Negative	N/A
Subulo gouazoubira	155M	Negative	0	0	Negative	N/A
Subulo gouazoubira	156M	Negative	0	0	Negative	N/A
Subulo gouazoubira	180M	Negative	0	0	Negative	N/A
Desmodus rotundus	13MO	Negative	0	0	Negative	N/A
Desmodus rotundus	24MO	Positive	13	5.079	Positive	30.2
Desmodus rotundus	25MO	Positive	31	11.79	Negative	N/A
Desmodus rotundus	33MO	Positive	189	72.42	Negative	N/A
Desmodus rotundus	34MO	Positive	10	3.900	Negative	N/A
Desmodus rotundus	40MO	Positive	98	38.81	Negative	N/A
Desmodus rotundus	50MO	Positive	233	93.61	Positive	27.81
Desmodus rotundus	58MO	Positive	13	5.152	Negative	N/A
Desmodus rotundus	65MO	Positive	1	0.398	Negative	N/A

Desmodus rotundus	68MO	Positive	1	0.405	Negative	N/A
Cerdocyon thous	1L	Negative	0	0	Negative	N/A
Leopardus pardalis	4L	Negative	0	0	Negative	N/A
Leopardus pardalis	5L	Negative	0	0	Negative	N/A
Leopardus pardalis	11L	Negative	0	0	Negative	N/A
Leopardus pardalis	14L	Negative	0	0	Negative	N/A
Leopardus pardalis	20L	Negative	0	0	Negative	N/A
Leopardus pardalis	21L	Negative	0	0	Negative	N/A
Panthera onca	2P	Positive	2	0.805	Negative	N/A
Panthera onca	3P	Negative	0	0	Negative	N/A
Panthera onca	4P	Positive	1	0.402	Negative	N/A
Panthera onca	5P	Positive	1	0.409	Negative	N/A
Panthera onca	7P	Negative	0	0	Negative	N/A
Panthera onca	9P	Negative	0	0	Negative	N/A
Panthera onca	11P	Negative	0	0	Negative	N/A
Panthera onca	12P	Negative	0	0	Negative	N/A
Panthera onca	13P	Negative	0	0	Negative	N/A
Panthera onca	14P	Negative	0	0	Negative	N/A
Myrmecophaga tridactyla	51T	Negative	0	0	Negative	N/A
Myrmecophaga tridactyla	64T	Negative	0	0	Negative	N/A
Tamandua tetradactyla	66T	Negative	0	0	Negative	N/A
Myrmecophaga tridactyla	69T	Negative	0	0	Negative	N/A
Tamandua tetradactyla	72T	Negative	0	0	Negative	N/A
Myrmecophaga tridactyla	01T	Negative	0	0	Negative	N/A
Myrmecophaga tridactyla	03T	Negative	0	0	Negative	N/A
Myrmecophaga tridactyla	05T	Negative	0	0	Positive	39.02
Myrmecophaga tridactyla	06T	Negative	0	0	Negative	N/A
Tamandua tetradactyla	12T	Negative	0	0	Negative	N/A
Pseudoseisura unirufa	18C	Negative	0	0	Negative	N/A
Saltator coerulescens	27C	Negative	0	0	Negative	N/A
Turdus leocomelas	97C	Positive	1	0.398	Negative	N/A
Cercomacra melanaria	98C	Negative	0	0	Negative	N/A
Ramphocelus carbo	404C	Negative	0	0	Negative	N/A
Basileuterus flaveolus	405C	Negative	0	0	Negative	N/A
Saltator coerulescens	452C	Positive	1	0.399	Negative	N/A
Leptotila verreauxi	453C	Negative	0	0	Negative	N/A
Tapirus terrestris	1A	Negative	0	0	Negative	N/A
Tapirus terrestris	2A	Positive	1	0.390	Negative	N/A
Tapirus terrestris	10A	Negative	0	0	Negative	N/A
Tapirus terrestris	11A	Positive	1	0.391	Negative	N/A
Tapirus terrestris	63A	Negative	0	0	Negative	N/A
Tapirus terrestris	64A	Negative	0	0	Negative	N/A

Tapirus terrestris	75A	Negative	0	0	Negative	N/A
Tapirus terrestris	82A	Negative	0	0	Negative	N/A
Tapirus terrestris	90A	Negative	0	0	Negative	N/A
Tapirus terrestris	110A	Negative	0	0	Negative	N/A
Tapirus terrestris	112A	Negative	0	0	Negative	N/A
Tapirus terrestris	114A	Negative	0	0	Negative	N/A
Tapirus terrestris	118A	Negative	0	0	Negative	N/A
Tapirus terrestris	119A	Positive	2	0.754	Negative	N/A
Desmodus rotundus	176MO	Positive	1	0.369	Negative	N/A
Desmodus rotundus	182MO	Positive	3869	4150.8	Positive	24.64
Desmodus rotundus	214MO	Negative	0	0	Negative	N/A
Desmodus rotundus	215MO	Positive	7	2.656	Negative	N/A
Desmodus rotundus	216MO	Positive	1	0.357	Negative	N/A
Desmodus rotundus	218MO	Positive	107	40.79	Positive	30.92
Desmodus rotundus	219MO	Positive	59	22.76	Negative	N/A
Desmodus rotundus	220MO	Positive	96	36.38	Positive	33.74
Desmodus rotundus	221MO	Positive	79	29.87	Positive	29.79
Desmodus rotundus	231MO	Positive	49	18.54	Positive	32.59
Desmodus rotundus	205MO	Positive	381	145.5	Negative	N/A
Desmodus rotundus	19MO	Positive	461	626.1	Positive	12.26
Desmodus rotundus	27MO	Positive	839	572.6	Positive	28.25
Desmodus rotundus	35MO	Positive	128	48.87	Positive	30.21
Desmodus rotundus	76MO	Positive	442	1038.9	Positive	25.75
Desmodus rotundus	82MO	Negative	0	0	Negative	N/A
Desmodus rotundus	115MO	Positive	5	1.958	Negative	N/A
Desmodus rotundus	90MO	Positive	27	10.55	Negative	N/A
Desmodus rotundus	170MO	Positive	37	14.44	Negative	N/A
Desmodus rotundus	179MO	Positive	46	18.21	Positive	31.59
Desmodus rotundus	207MO	Positive	17	6.641	Negative	N/A
Desmodus rotundus	63MO	Positive	17	6.641	Negative	N/A
Desmodus rotundus	70MO	Positive	1	0.356	Negative	N/A
Desmodus rotundus	88MO	Positive	2	0.753	Negative	N/A
Desmodus rotundus	92MO	Positive	7	2.646	Negative	N/A
Leopardus pardalis	9L	Negative	0	0	Negative	N/A
Panthera onca	15P	Negative	0	0	Positive	42.86
Panthera onca	16P	Negative	0	0	Negative	N/A
Panthera onca	17P	Positive	1	0.395	Negative	N/A
Panthera onca	19P	Negative	0	0	Negative	N/A
Panthera onca	20P	Negative	0	0	Negative	N/A
Panthera onca	21P	Negative	0	0	Negative	N/A
Panthera onca	22P	Negative	0	0	Negative	N/A
Panthera onca	23P	Negative	0	0	Negative	N/A
Panthera onca	24P	Negative	0	0	Negative	N/A
Panthera onca	25P	Positive	3	1.135	Negative	N/A
Myrmecophaga tridactyla	17T	Negative	0	0.756	Negative	N/A
Euphractus sexcinctus	32T	Negative	0	0	Negative	N/A

Myrmecophaga tridactyla	50T	Negative	0	0	Negative	N/A
Dasypus novemcinctus	76T	Negative	0	0	Positive	44.7
Dasypus novemcinctus	79T	Negative	0	0	Positive	13.03
Myrmecophaga tridactyla	81T	Negative	0	0	Negative	N/A
Dasypus novemcinctus	149T	Positive	1	0.404	Negative	N/A
Tapirus terrestris	122A	Negative	0	0	Negative	N/A
Tapirus terrestris	124A	Negative	0	0	Negative	N/A
Subulo gouazoubira	196M	Positive	1	0.392	Negative	N/A
Mazama jucunda	204M	Positive	1	0.390	Negative	N/A
Mazama jucunda	208M	Positive	5	1.909	Negative	N/A
Subulo gouazoubira	209M	Negative	0	0	Negative	N/A
Mazama jucunda	210M	Negative	0	0	Negative	N/A
Mazama rufa	267M	Negative	0	0	Negative	N/A
Mazama rufa	268M	Negative	0	0	Negative	N/A
Subulo gouazoubira	298M	Positive	3	1.216	Negative	N/A
Subulo gouazoubira	299M	Negative	0	0	Negative	N/A
Subulo gouazoubira	300M	Positive	3	1.225	Positive	33.26
Subulo gouazoubira	301M	Positive	1	0.405	Negative	N/A
Subulo gouazoubira	302M	Positive	1	0.400	Positive	31.03
Subulo gouazoubira	303M	Negative	0	0	Negative	N/A
Subulo gouazoubira	307M	Positive	1	0.394	Negative	N/A
Subulo gouazoubira	313M	Negative	0	0	Negative	N/A
Subulo gouazoubira	314M	Negative	0	0	Negative	N/A
Subulo gouazoubira	315M	Negative	0	0	Negative	N/A
Subulo gouazoubira	316M	Negative	0	0	Negative	N/A
Subulo gouazoubira	317M	Negative	0	0	Negative	N/A
Subulo gouazoubira	318M	Negative	0	0	Negative	N/A
Subulo gouazoubira	319M	Positive	1	0.401	Negative	N/A
Subulo gouazoubira	320M	Negative	0	0	Negative	N/A
Blastocerus dichotomus	35B	Negative	0	0	Negative	N/A
Blastocerus dichotomus	71B	Positive	1	0.399	Negative	N/A
Blastocerus dichotomus	78B	Negative	0	0	Negative	N/A
Blastocerus dichotomus	79B	Negative	0	0	Negative	N/A
Blastocerus dichotomus	94B	Negative	0	0	Negative	N/A
Blastocerus dichotomus	104B	Positive	2	0.759	Negative	N/A
Blastocerus dichotomus	24B	Positive	4	1.561	Negative	N/A
Blastocerus dichotomus	25B	Positive	163	64.43	Negative	N/A
Blastocerus dichotomus	26B	Positive	183	72.37	Positive	13.74
Blastocerus dichotomus	28B	Positive	65	25.91	Negative	N/A
Blastocerus dichotomus	29B	Positive	7	2.798	Negative	N/A
Blastocerus dichotomus	37B	Negative	0	0	Negative	N/A
Blastocerus dichotomus	38B	Positive	1	0.394	Negative	N/A
Blastocerus dichotomus	40B	Negative	0	0	Positive	31.07
Blastocerus dichotomus	41B	Negative	0	0	Positive	34.9
Blastocerus dichotomus	43B	Negative	0	0	Negative	N/A

Blastocerus dichotomus	45B	Positive	1	0.388	Negative	N/A
Blastocerus dichotomus	46B	Positive	3	1.113	Negative	N/A
Blastocerus dichotomus	48B	Negative	0	0	Positive	15.91
Blastocerus dichotomus	49B	Negative	0	0	Negative	N/A
Blastocerus dichotomus	51B	Positive	10	3.775	Positive	14.62
Blastocerus dichotomus	52B	Positive	1	0.388	Negative	N/A
Blastocerus dichotomus	55B	Positive	1	0.383	Positive	42.86
Blastocerus dichotomus	64B	Positive	2	0.760	Negative	N/A
Blastocerus dichotomus	68B	Negative	0	0	Negative	N/A
Blastocerus dichotomus	76B	Positive	372	160.9	Positive	19.04
Desmodus rotundus	36MO	Positive	544	202.3	Negative	N/A
Desmodus rotundus	43MO	Positive	1	0.366	Negative	N/A
Desmodus rotundus	44MO	Negative	0	0	Negative	N/A
Desmodus rotundus	47MO	Negative	0	0	Negative	N/A
Desmodus rotundus	54MO	Positive	56	20.30	Negative	N/A
Blastocerus dichotomus	77B	Positive	5	1.801	Negative	N/A
Blastocerus dichotomus	78B	Negative	0	0	Negative	N/A
Blastocerus dichotomus	80B	Positive	4	1.453	Negative	N/A
Blastocerus dichotomus	81B	Positive	1	0.356	Negative	N/A
Blastocerus dichotomus	83B	Positive	12	4.379	Negative	N/A
Blastocerus dichotomus	84B	Positive	4	1.447	Negative	N/A
Blastocerus dichotomus	86B	Positive	26	9.500	Negative	N/A
Blastocerus dichotomus	87B	Positive	4	1.424	Negative	N/A
Blastocerus dichotomus	88B	Negative	0	0	Negative	N/A
Blastocerus dichotomus	92B	Positive	2	0.714	Negative	N/A
Blastocerus dichotomus	93B	Positive	251	91.81	Negative	N/A
Blastocerus dichotomus	98B	Positive	10	4.526	Negative	N/A
Blastocerus dichotomus	100B	Negative	0	0	Negative	N/A
Blastocerus dichotomus	102B	Negative	0	0	Negative	N/A
Blastocerus dichotomus	103B	Negative	0	0	Negative	N/A
Blastocerus dichotomus	106B	Negative	0	0	Negative	N/A
Blastocerus dichotomus	107B	Negative	0	0	Negative	N/A
Blastocerus dichotomus	108B	Negative	0	0	Negative	N/A
Blastocerus dichotomus	110B	Positive	5	1.783	Negative	N/A
Blastocerus dichotomus	112B	Positive	66	23.62	Negative	N/A
Blastocerus dichotomus	128B	Negative	0	0	Positive	43.51
Blastocerus dichotomus	130B	Negative	0	0	Negative	N/A
Blastocerus dichotomus	131B	Positive	147	52.80	Negative	N/A
Blastocerus dichotomus	134B	Positive	8	2.919	Negative	N/A
Blastocerus dichotomus	136B	Positive	47	16.77	Negative	N/A
Blastocerus dichotomus	138B	Negative	0	0	Negative	N/A
Blastocerus dichotomus	25B1	Negative	0	0	Negative	N/A
Blastocerus dichotomus	50B1	Positive	1	0.357	Negative	N/A
Blastocerus dichotomus	58B1	Negative	0	0	Negative	N/A
Blastocerus dichotomus	65B1	Negative	0	0	Negative	N/A
Blastocerus dichotomus	74B1	Negative	0	0	Negative	N/A

Blastocerus dichotomus	82B1	Negative	0	0	Negative	N/A
Blastocerus dichotomus	95B1	Negative	0	0	Negative	N/A
Blastocerus dichotomus	129B1	Negative	0	0	Negative	N/A
Blastocerus dichotomus	101B1	Negative	0	0	Negative	N/A
Blastocerus dichotomus	32B2	Negative	0	0	Negative	N/A
Blastocerus dichotomus	72B2	Negative	0	0	Negative	N/A
Blastocerus dichotomus	85B2	Negative	0	0	Negative	N/A
Blastocerus dichotomus	97B2	Negative	0	0	Negative	N/A
Blastocerus dichotomus	109B2	Negative	0	0	Negative	N/A
Blastocerus dichotomus	113B2	Negative	0	0	Negative	N/A
Blastocerus dichotomus	114B2	Negative	0	0	Negative	N/A
Blastocerus dichotomus	115B2	Positive	1	0.360	Negative	N/A
Blastocerus dichotomus	116B2	Negative	0	0	Negative	N/A
Blastocerus dichotomus	117B2	Negative	0	0	Negative	N/A
Blastocerus dichotomus	118B2	Negative	0	0	Negative	N/A
Blastocerus dichotomus	122B2	Negative	0	0	Negative	N/A
Blastocerus dichotomus	201B3	Negative	0	0	Negative	N/A
Blastocerus dichotomus	205B3	Negative	0	0	Negative	N/A
Blastocerus dichotomus	209B3	Positive	1	0.356	Negative	N/A
Blastocerus dichotomus	207B3	Negative	0	0	Negative	N/A
Blastocerus dichotomus	208B3	Positive	1	0.358	Negative	N/A
Blastocerus dichotomus	206B3	Negative	0	0	Positive	41.01
Blastocerus dichotomus	212B3	Positive	1	0.357	Negative	N/A
Blastocerus dichotomus	213B3	Negative	0	0	Negative	N/A
Blastocerus dichotomus	208MO	Positive	459	168.0	Positive	28.24
Blastocerus dichotomus	217B3	Negative	0	0	Negative	N/A
Blastocerus dichotomus	218B3	Positive	1	0.358	Negative	N/A
Blastocerus dichotomus	227B3	Negative	0	0	Negative	N/A
Blastocerus dichotomus	228B3	Negative	0	0	Negative	N/A
Blastocerus dichotomus	231B3	Negative	0	0	Negative	N/A
Blastocerus dichotomus	237B3	Negative	0	0	Negative	N/A
Blastocerus dichotomus	240B3	Negative	0	0	Negative	N/A
Blastocerus dichotomus	245B3	Negative	0	0	Negative	N/A
Blastocerus dichotomus	246B3	Negative	0	0	Negative	N/A
Blastocerus dichotomus	234B3	Positive	1	0.357	Negative	N/A
Blastocerus dichotomus	284B3	Negative	0	0	Negative	N/A
Blastocerus dichotomus	139B3	Negative	0	0	Negative	N/A
Blastocerus dichotomus	236B3	Negative	0	0	Negative	N/A
Blastocerus dichotomus	264B3	Negative	0	0	Negative	N/A
Blastocerus dichotomus	67B2	Negative	0	0	Negative	N/A
Desmodus rotundus	202MO	Negative	0	0	Negative	N/A
Cerdocyon thous	3L	Positive	2	0.712	Positive	38.02
Leopardus pardalis	7L	Negative	0	0	Negative	N/A
Cerdocyon thous	10L	Negative	0	0	Negative	N/A
Cerdocyon thous	13L	Negative	0	0	Negative	N/A
Cerdocyon thous	15L	Negative	0	0	Negative	N/A

Leopardus pardalis	16L	Positive	1	0.356	Negative	N/A
Cerdocyon thous	17L	Positive	1	0.357	Negative	N/A
Cerdocyon thous	19L	Positive	2	0.713	Negative	N/A
Leopardus pardalis	23L	Negative	0	0	Negative	N/A
Cerdocyon thous	24L	Negative	0	0	Negative	N/A
Cerdocyon thous	26L	Negative	0	0	Negative	N/A
Puma concolor	06P	Negative	0	0	Negative	N/A
Puma concolor	08P	Negative	0	0	Negative	N/A
Puma concolor	18P	Negative	0	0	Negative	N/A
Myrmecophaga tridactyla	97T	Negative	0	0	Negative	N/A
Blastocerus dichotomus	21B	Negative	0	0	Negative	N/A
Blastocerus dichotomus	23B	Negative	0	0	Negative	N/A
Blastocerus dichotomus	25B	Negative	0	0	Negative	N/A
Blastocerus dichotomus	27B	Negative	0	0	Negative	N/A
Blastocerus dichotomus	30B	Positive	1	0.361	Negative	N/A
Blastocerus dichotomus	31B	Negative	0	0	Negative	N/A
Blastocerus dichotomus	32B	Negative	0	0	Negative	N/A
Blastocerus dichotomus	33B	Negative	0	0	Negative	N/A
Blastocerus dichotomus	34B	Negative	0	0	Negative	N/A
Blastocerus dichotomus	36B	Negative	0	0	Negative	N/A
Blastocerus dichotomus	42B	Negative	0	0	Negative	N/A
Blastocerus dichotomus	44B	Negative	0	0	Negative	N/A
Blastocerus dichotomus	53B	Positive	1	0.356	Negative	N/A
Blastocerus dichotomus	54B	Negative	0	0	Negative	N/A
Blastocerus dichotomus	57B	Negative	0	0	Negative	N/A
Blastocerus dichotomus	59B	Negative	0	0	Negative	N/A
Blastocerus dichotomus	60B	Negative	0	0	Negative	N/A
Blastocerus dichotomus	66B	Negative	0	0	Negative	N/A
Blastocerus dichotomus	89B	Negative	0	0	Negative	N/A
Blastocerus dichotomus	90B	Negative	0	0	Negative	N/A
Blastocerus dichotomus	99B	Negative	0	0	Negative	N/A
Blastocerus dichotomus	105B	Negative	0	0	Negative	N/A
Blastocerus dichotomus	111B	Negative	0	0	Negative	N/A
Blastocerus dichotomus	113B	Negative	0	0	Negative	N/A
Blastocerus dichotomus	117B	Negative	0	0	Negative	N/A
Blastocerus dichotomus	123B	Negative	0	0	Negative	N/A
Blastocerus dichotomus	124B	Positive	1	0.361	Negative	N/A
Blastocerus dichotomus	126B	Negative	0	0	Negative	N/A
Blastocerus dichotomus	127B	Negative	0	0	Negative	N/A
Blastocerus dichotomus	135B	Negative	0	0	Negative	N/A
Blastocerus dichotomus	160B	Negative	0	0	Negative	N/A
Blastocerus dichotomus	229B	Negative	0	0	Negative	N/A
Blastocerus dichotomus	230B	Positive	1	0.357	Negative	N/A
Blastocerus dichotomus	235B	Negative	0	0	Negative	N/A
Blastocerus dichotomus	238B	Negative	0	0	Negative	N/A
Blastocerus dichotomus	241B	Negative	0	0	Negative	N/A

Blastocerus dichotomus	265B	Negative	0	0	Negative	N/A
Tapirus terrestris	3A	Negative	0	0	Negative	N/A
Tapirus terrestris	5A	Negative	0	0	Negative	N/A
Tapirus terrestris	6A	Negative	0	0	Negative	N/A
Tapirus terrestris	21A	Negative	0	0	Negative	N/A
Tapirus terrestris	95A	Positive	1	0.361	Negative	N/A
Tapirus terrestris	106A	Negative	0	0	Negative	N/A
Tapirus terrestris	104A	Negative	0	0	Negative	N/A
Tapirus terrestris	107A	Negative	0	0	Negative	N/A
Myrmecophaga tridactyla	163T	Negative	0	0	Negative	N/A
Myrmecophaga tridactyla	172T	Negative	0	0	Negative	N/A
Myrmecophaga tridactyla	202T	Negative	0	0	Negative	N/A
Myrmecophaga tridactyla	178T	Negative	0	0	Negative	N/A
Euphractus sexcinctus	100T	Positive	3	1.080	Positive	40.61
Euphractus sexcinctus	35T	Negative	0	0	Negative	N/A
Euphractus sexcinctus	25T	Positive	2	0.720	Negative	N/A
Tamandua tetradactyla	101T	Positive	1	0.360	Negative	N/A
Tamandua tetradactyla	133T	Negative	0	0	Negative	N/A
Tamandua tetradactyla	115T	Negative	0	0	Negative	N/A
Tamandua tetradactyla	57T	Negative	0	0	Negative	N/A
Tamandua tetradactyla	73T	Negative	0	0	Negative	N/A
Priodontes maximus	135T	Negative	0	0	Negative	N/A
Priodontes maximus	145T	Negative	0	0	Negative	N/A
Euphractus sexcinctus	150T	Positive	1	0.360	Negative	N/A
Priodontes maximus	154T	Negative	0	0	Negative	N/A
Dasypus novemcinctus	41T	Positive	2	0.714	Negative	N/A
Dasypus novemcinctus	42T	Negative	0	0	Negative	N/A
Dasypus novemcinctus	88T	Negative	0	0	Negative	N/A
Oecomys mamorae	58R	Positive	214	77.40	Negative	N/A
Oecomys mamorae	18R	Positive	31	11.21	Negative	N/A
Oecomys mamorae	48R	Positive	11	3.932	Negative	N/A
Oecomys mamorae	84R	Negative	0	0	Negative	N/A
Thrichomys fosteri	7R	Negative	0	0	Negative	N/A
Clyomis laticeps	42R	Negative	0	0	Negative	N/A
Thrichomys fosteri	43R	Positive	1721	694.1	Positive	31.21
Thrichomys fosteri	13R	Negative	0	0	Negative	N/A
Thrichomys fosteri	60R	Negative	0	0	Negative	N/A
Leptotila verreauxi	107C	Negative	0	0	Positive	43.84
Icterus cayanensis	72C	Negative	0	0	Negative	N/A
Lepidocolaptes angustirostris	59C	Negative	0	0	Negative	N/A
Leptotila verreauxi	78C	Negative	0	0	Negative	N/A
Ramphocelus carbo	90C	Negative	0	0	Negative	N/A
Turdus leocomelas	85C	Negative	0	0	Negative	N/A

Fumarius rufus	119C	Negative	0	0	Negative	N/A
Saltator coerulescens	120C	Positive	1	0.356	Negative	N/A
Furnarius leucopus	117C	Negative	0	0	Negative	N/A
Cyanocorax chryrops	124C	Positive	1	0.356	Negative	N/A
Desmodus rotundus	162MO	Positive	11	3.922	Positive	1.87
Desmodus rotundus	180MO	Positive	951	359.8	Negative	N/A
Desmodus rotundus	158MO	Positive	536	197.3	Positive	29.59
Desmodus rotundus	223MO	Positive	1	0.357	Positive	36.56
Desmodus rotundus	200MO	Positive	2	0.713	Negative	N/A
Desmodus rotundus	224MO	Positive	15	5.355	Positive	36.19
Desmodus rotundus	222MO	Positive	4	1.429	Negative	N/A
Desmodus rotundus	201MO	Positive	139	49.98	Negative	N/A

Table S2: Results obtained for each test carried out for *Borrelia* spp.

		dPCR			qPCR	
**Animal species**	**ID**	**Result**	**Positive Partitions**	**Conc (copies/µL)**	**Result**	**CQ**
Subulo gouazoubira	182M	Positive	1	0.413	Negative	N/A
Mazama jucunda	215M	Positive	1	0.378	Negative	N/A
Mazama rufa	270M	Positive	2	0.759	Negative	N/A
Blastocerus dichotomus	63B	Negative	0	0	Negative	N/A
Blastocerus dichotomus	75B	Negative	0	0	Negative	N/A
Blastocerus dichotomus	120B2	Negative	0	0	Negative	N/A
Blastocerus dichotomus	121B2	Negative	0	0	Negative	N/A
Blastocerus dichotomus	222B3	Negative	0	0	Negative	N/A
Blastocerus dichotomus	226B3	Negative	0	0	Negative	N/A
Desmodus rotundus	91MO	Negative	0	0	Negative	N/A
Desmodus rotundus	8MO	Positive	1	0.375	Negative	N/A
Cerdocyon thous	2L	Negative	0	0	Negative	N/A
Leopardus pardalis	8L	Negative	0	0	Negative	N/A
Leopardus pardalis	22L	Negative	0	0	Negative	N/A
Cerdocyon thous	25L	Negative	0	0	Negative	N/A
Myrmecophaga tridactyla	59T	Negative	0	0	Negative	N/A
Myrmecophaga tridactyla	62T	Negative	0	0	Negative	N/A
Tamandua tetradactyla	71T	Negative	0	0	Negative	N/A
Dasypus novemcinctus	16T	Negative	0	0	Negative	N/A
Priodontes maximus	153T	Negative	0	0	Negative	N/A
Priodontes maximus	159T	Negative	0	0	Negative	N/A
Priodontes maximus	160T	Negative	0	0	Negative	N/A
Ozotocerus bezoarticus	06O	Negative	0	0	Negative	N/A
Ozotocerus bezoarticus	07O	Negative	0	0	Negative	N/A
Ozotocerus bezoarticus	08O	Negative	0	0	Negative	N/A
Ozotocerus bezoarticus	09O	Negative	0	0	Negative	N/A
Ozotocerus bezoarticus	10O	Negative	0	0	Negative	N/A
Subulo gouazoubira	154M	Negative	0	0	Negative	N/A
Subulo gouazoubira	155M	Negative	0	0	Negative	N/A
Subulo gouazoubira	156M	Negative	0	0	Negative	N/A
Subulo gouazoubira	180M	Negative	0	0	Negative	N/A
Desmodus rotundus	13MO	Negative	0	0	Negative	N/A
Desmodus rotundus	24MO	Positive	2	0.781	Negative	N/A
Desmodus rotundus	25MO	Negative	0	0	Negative	N/A
Desmodus rotundus	33MO	Negative	0	0	Negative	N/A
Desmodus rotundus	34MO	Positive	1	0.390	Negative	N/A
Desmodus rotundus	40MO	Negative	0	0	Negative	N/A
Desmodus rotundus	50MO	Negative	0	0	Negative	N/A
Desmodus rotundus	58MO	Positive	1	0.396	Negative	N/A
Desmodus rotundus	65MO	Positive	1	0.398	Negative	N/A
Desmodus rotundus	68MO	Negative	0	0	Negative	N/A
Cerdocyon thous	1L	Positive	1	0.401	Negative	N/A
Leopardus pardalis	4L	Negative	0	0	Negative	N/A
Leopardus pardalis	5L	Negative	0	0	Negative	N/A
Leopardus pardalis	11L	Negative	0	0	Negative	N/A
Leopardus pardalis	14L	Negative	0	0	Negative	N/A
Leopardus pardalis	20L	Negative	0	0	Negative	N/A
Leopardus pardalis	21L	Negative	0	0	Negative	N/A
Panthera onca	2P	Positive	1	0.402	Negative	N/A

Panthera onca	3P	Positive	3	1.219	Negative	N/A
Panthera onca	4P	Negative	0	0	Negative	N/A
Panthera onca	5P	Positive	1	0.409	Negative	N/A
Panthera onca	7P	Negative	0	0	Negative	N/A
Panthera onca	9P	Negative	0	0	Negative	N/A
Panthera onca	11P	Positive	1	0.401	Negative	N/A
Panthera onca	12P	Positive	2	0.789	Negative	N/A
Panthera onca	13P	Negative	0	0	Negative	N/A
Panthera onca	14P	Negative	0	0	Negative	N/A
Myrmecophaga tridactyla	51T	Negative	0	0	Negative	N/A
Myrmecophaga tridactyla	64T	Negative	0	0	Negative	N/A
Tamandua tetradactyla	66T	Positive	1	0.403	Negative	N/A
Myrmecophaga tridactyla	69T	Negative	0	0	Negative	N/A
Tamandua tetradactyla	72T	Negative	0	0	Negative	N/A
Myrmecophaga tridactyla	01T	Negative	0	0	Negative	N/A
Myrmecophaga tridactyla	03T	Positive	1	0.401	Negative	N/A
Myrmecophaga tridactyla	05T	Negative	0	0	Negative	N/A
Myrmecophaga tridactyla	06T	Negative	0	0	Negative	N/A
Tamandua tetradactyla	12T	Negative	0	0	Negative	N/A
Pseudoseisura unirufa	18C	Negative	0	0	Negative	N/A
Saltator coerulescens	27C	Negative	0	0	Negative	N/A
Turdus leocomelas	97C	Negative	0	0	Negative	N/A
Cercomacra melanaria	98C	Negative	0	0	Negative	N/A
Ramphocelus carbo	404C	Negative	0	0	Negative	N/A
Basileuterus flaveolus	405C	Negative	0	0	Negative	N/A
Saltator coerulescens	452C	Negative	0	0	Negative	N/A
Leptotila verreauxi	453C	Negative	0	0	Negative	N/A
Tapirus terrestris	1A	Negative	0	0	Negative	N/A
Tapirus terrestris	2A	Positive	1	0.390	Negative	N/A
Tapirus terrestris	10A	Negative	0	0	Negative	N/A
Tapirus terrestris	11A	Negative	0	0	Negative	N/A
Tapirus terrestris	63A	Negative	0	0	Negative	N/A
Tapirus terrestris	64A	Negative	0	0	Negative	N/A
Tapirus terrestris	75A	Positive	2	1.052	Negative	N/A
Tapirus terrestris	82A	Negative	0	0	Negative	N/A
Tapirus terrestris	90A	Negative	0	0	Negative	N/A
Tapirus terrestris	110A	Negative	0	0	Negative	N/A
Tapirus terrestris	112A	Negative	0	0	Negative	N/A
Tapirus terrestris	114A	Positive	1	0.383	Negative	N/A
Tapirus terrestris	118A	Negative	0	0	Negative	N/A
Tapirus terrestris	119A	Negative	0	0	Negative	N/A
Desmodus rotundus	176MO	Positive	1	0.369	Positive	43.26
Desmodus rotundus	182MO	Negative	0	0	Negative	N/A
Desmodus rotundus	214MO	Negative	0	0	Negative	N/A
Desmodus rotundus	215MO	Negative	0	0	Negative	N/A
Desmodus rotundus	216MO	Negative	0	0	Negative	N/A
Desmodus rotundus	218MO	Negative	0	0	Negative	N/A
Desmodus rotundus	219MO	Negative	0	0	Positive	44.93
Desmodus rotundus	220MO	Positive	2	0.754	Negative	N/A
Desmodus rotundus	221MO	Positive	2	0.753	Negative	N/A
Desmodus rotundus	231MO	Positive	1	0.377	Negative	N/A
Desmodus rotundus	205MO	Negative	0	0	Negative	N/A
Desmodus rotundus	19MO	Negative	0	0	Negative	N/A
Desmodus rotundus	27MO	Negative	0	0	Negative	N/A

Desmodus rotundus	35MO	Negative	0	0	Negative	N/A
Desmodus rotundus	76MO	Positive	1	0.386	Negative	N/A
Desmodus rotundus	82MO	Negative	0	0	Negative	N/A
Desmodus rotundus	115MO	Negative	0	0	Negative	N/A
Desmodus rotundus	90MO	Negative	0	0	Negative	N/A
Desmodus rotundus	170MO	Negative	0	0	Negative	N/A
Desmodus rotundus	179MO	Negative	0	0	Negative	N/A
Desmodus rotundus	207MO	Negative	0	0	Negative	N/A
Desmodus rotundus	63MO	Negative	0	0	Negative	N/A
Desmodus rotundus	70MO	Negative	0	0	Negative	N/A
Desmodus rotundus	88MO	Negative	0	0	Negative	N/A
Desmodus rotundus	92MO	Positive	1	0.378	Negative	N/A
Leopardus pardalis	9L	Negative	0	0	Negative	N/A
Panthera onca	15P	Negative	0	0	Negative	N/A
Panthera onca	16P	Negative	0	0	Negative	N/A
Panthera onca	17P	Positive	1	0.395	Negative	N/A
Panthera onca	19P	Negative	0	0	Negative	N/A
Panthera onca	20P	Negative	0	0	Negative	N/A
Panthera onca	21P	Negative	0	0	Negative	N/A
Panthera onca	22P	Negative	0	0	Negative	N/A
Panthera onca	23P	Negative	0	0	Negative	N/A
Panthera onca	24P	Negative	0	0	Negative	N/A
Panthera onca	25P	Negative	0	0	Negative	N/A
Myrmecophaga tridactyla	17T	Negative	0	0	Negative	N/A
Euphractus sexcinctus	32T	Negative	0	0	Negative	N/A
Myrmecophaga tridactyla	50T	Negative	0	0	Negative	N/A
Dasypus novemcinctus	76T	Negative	0	0	Negative	N/A
Dasypus novemcinctus	79T	Negative	0	0	Negative	N/A
Myrmecophaga tridactyla	81T	Negative	0	0	Negative	N/A
Dasypus novemcinctus	149T	Negative	0	0	Negative	N/A
Tapirus terrestris	122A	Negative	0	0	Negative	N/A
Tapirus terrestris	124A	Negative	0	0	Negative	N/A
Subulo gouazoubira	196M	Positive	2	0.784	Negative	N/A
Mazama jucunda	204M	Positive	1	0.390	Negative	N/A
Mazama jucunda	208M	Positive	1	0.381	Negative	N/A
Subulo gouazoubira	209M	Negative	0	0	Negative	N/A
Mazama jucunda	210M	Negative	0	0	Negative	N/A
Mazama rufa	267M	Negative	0	0	Negative	N/A
Mazama rufa	268M	Negative	0	0	Negative	N/A
Subulo gouazoubira	298M	Positive	1	0.405	Negative	N/A
Subulo gouazoubira	299M	Negative	0	0	Negative	N/A
Subulo gouazoubira	300M	Negative	0	0	Negative	N/A
Subulo gouazoubira	301M	Positive	1	0.405	Negative	N/A
Subulo gouazoubira	302M	Negative	0	0	Negative	N/A
Subulo gouazoubira	303M	Negative	0	0	Negative	N/A
Subulo gouazoubira	307M	Negative	0	0	Negative	N/A
Subulo gouazoubira	313M	Negative	0	0	Negative	N/A
Subulo gouazoubira	314M	Positive	1	0.385	Negative	N/A
Subulo gouazoubira	315M	Negative	0	0	Negative	N/A
Subulo gouazoubira	316M	Negative	0	0	Negative	N/A
Subulo gouazoubira	317M	Negative	0	0	Negative	N/A
Subulo gouazoubira	318M	Negative	0	0	Negative	N/A
Subulo gouazoubira	319M	Negative	0	0	Negative	N/A
Subulo gouazoubira	320M	Positive	1	0.405	Negative	N/A

Blastocerus dichotomus	35B	Positive	1	0.400	Negative	N/A
Blastocerus dichotomus	71B	Negative	0	0	Negative	N/A
Blastocerus dichotomus	78B	Negative	0	0	Negative	N/A
Blastocerus dichotomus	79B	Negative	0	0	Negative	N/A
Blastocerus dichotomus	94B	Negative	0	0	Negative	N/A
Blastocerus dichotomus	104B	Positive	1	0.379	Negative	N/A
Blastocerus dichotomus	24B	Negative	0	0	Negative	N/A
Blastocerus dichotomus	25B	Negative	0	0	Negative	N/A
Blastocerus dichotomus	26B	Negative	0	0	Negative	N/A
Blastocerus dichotomus	28B	Negative	0	0	Negative	N/A
Blastocerus dichotomus	29B	Negative	0	0	Negative	N/A
Blastocerus dichotomus	37B	Negative	0	0	Negative	N/A
Blastocerus dichotomus	38B	Negative	0	0	Negative	N/A
Blastocerus dichotomus	40B	Negative	0	0	Negative	N/A
Blastocerus dichotomus	41B	Negative	0	0	Negative	N/A
Blastocerus dichotomus	43B	Negative	0	0	Negative	N/A
Blastocerus dichotomus	45B	Negative	0	0	Negative	N/A
Blastocerus dichotomus	46B	Negative	0	0	Negative	N/A
Blastocerus dichotomus	48B	Negative	0	0	Negative	N/A
Blastocerus dichotomus	49B	Positive	1	0.381	Negative	N/A
Blastocerus dichotomus	51B	Positive	1	0.377	Negative	N/A
Blastocerus dichotomus	52B	Negative	0	0	Negative	N/A
Blastocerus dichotomus	55B	Negative	0	0	Negative	N/A
Blastocerus dichotomus	64B	Positive	1	0.380	Negative	N/A
Blastocerus dichotomus	68B	Negative	0	0	Negative	N/A
Blastocerus dichotomus	76B	Positive	7	2.636	Negative	N/A
Desmodus rotundus	36MO	Positive	2	0.719	Negative	N/A
Desmodus rotundus	43MO	Negative	0	0	Negative	N/A
Desmodus rotundus	44MO	Negative	0	0	Negative	N/A
Desmodus rotundus	47MO	Negative	0	0	Negative	N/A
Desmodus rotundus	54MO	Negative	0	0	Negative	N/A
Blastocerus dichotomus	77B	Negative	0	0	Negative	N/A
Blastocerus dichotomus	78B	Positive	1	0.357	Negative	N/A
Blastocerus dichotomus	80B	Negative	0	0	Negative	N/A
Blastocerus dichotomus	81B	Negative	0	0	Negative	N/A
Blastocerus dichotomus	83B	Negative	0	0	Negative	N/A
Blastocerus dichotomus	84B	Negative	0	0	Negative	N/A
Blastocerus dichotomus	86B	Negative	0	0	Negative	N/A
Blastocerus dichotomus	87B	Negative	0	0	Negative	N/A
Blastocerus dichotomus	88B	Negative	0	0	Negative	N/A
Blastocerus dichotomus	92B	Negative	0	0	Negative	N/A
Blastocerus dichotomus	93B	Negative	0	0	Negative	N/A
Blastocerus dichotomus	98B	Negative	0	0	Negative	N/A
Blastocerus dichotomus	100B	Negative	0	0	Negative	N/A
Blastocerus dichotomus	102B	Negative	0	0	Negative	N/A
Blastocerus dichotomus	103B	Negative	0	0	Negative	N/A
Blastocerus dichotomus	106B	Negative	0	0	Negative	N/A
Blastocerus dichotomus	107B	Negative	0	0	Negative	N/A
Blastocerus dichotomus	108B	Negative	0	0	Negative	N/A
Blastocerus dichotomus	110B	Negative	0	0	Negative	N/A
Blastocerus dichotomus	112B	Negative	0	0	Negative	N/A
Blastocerus dichotomus	128B	Negative	0	0	Negative	N/A
Blastocerus dichotomus	130B	Negative	0	0	Negative	N/A
Blastocerus dichotomus	131B	Negative	0	0	Negative	N/A

Blastocerus dichotomus	134B	Negative	0	0	Negative	N/A
Blastocerus dichotomus	136B	Negative	0	0	Negative	N/A
Blastocerus dichotomus	138B	Negative	0	0	Negative	N/A
Blastocerus dichotomus	25B1	Negative	0	0	Negative	N/A
Blastocerus dichotomus	50B1	Negative	0	0	Negative	N/A
Blastocerus dichotomus	58B1	Negative	0	0	Negative	N/A
Blastocerus dichotomus	65B1	Negative	0	0	Negative	N/A
Blastocerus dichotomus	74B1	Negative	0	0	Negative	N/A
Blastocerus dichotomus	82B1	Negative	0	0	Negative	N/A
Blastocerus dichotomus	95B1	Negative	0	0	Negative	N/A
Blastocerus dichotomus	129B1	Negative	0	0	Negative	N/A
Blastocerus dichotomus	101B1	Negative	0	0	Negative	N/A
Blastocerus dichotomus	32B2	Negative	0	0	Negative	N/A
Blastocerus dichotomus	72B2	Negative	0	0	Negative	N/A
Blastocerus dichotomus	85B2	Negative	0	0	Negative	N/A
Blastocerus dichotomus	97B2	Negative	0	0	Negative	N/A
Blastocerus dichotomus	109B2	1	1	0.356	Negative	N/A
Blastocerus dichotomus	113B2	Negative	0	0	Negative	N/A
Blastocerus dichotomus	114B2	Negative	0	0	Negative	N/A
Blastocerus dichotomus	115B2	Negative	0	0	Negative	N/A
Blastocerus dichotomus	116B2	Negative	0	0	Negative	N/A
Blastocerus dichotomus	117B2	Negative	0	0	Negative	N/A
Blastocerus dichotomus	118B2	Negative	0	0	Negative	N/A
Blastocerus dichotomus	122B2	Negative	0	0	Negative	N/A
Blastocerus dichotomus	201B3	Negative	0	0	Negative	N/A
Blastocerus dichotomus	205B3	Negative	0	0	Negative	N/A
Blastocerus dichotomus	209B3	Positive	1	0.356	Negative	N/A
Blastocerus dichotomus	207B3	Negative	0	0	Negative	N/A
Blastocerus dichotomus	208B3	Negative	0	0	Negative	N/A
Blastocerus dichotomus	206B3	Negative	0	0	Negative	N/A
Blastocerus dichotomus	212B3	Negative	0	0	Negative	N/A
Blastocerus dichotomus	213B3	Negative	0	0	Negative	N/A
Blastocerus dichotomus	208MO	Negative	0	0	Negative	N/A
Blastocerus dichotomus	217B3	Negative	0	0	Negative	N/A
Blastocerus dichotomus	218B3	Negative	0	0	Negative	N/A
Blastocerus dichotomus	227B3	Negative	0	0	Negative	N/A
Blastocerus dichotomus	228B3	Negative	0	0	Negative	N/A
Blastocerus dichotomus	231B3	Negative	0	0	Negative	N/A
Blastocerus dichotomus	237B3	Negative	0	0	Negative	N/A
Blastocerus dichotomus	240B3	Negative	0	0	Negative	N/A
Blastocerus dichotomus	245B3	Negative	0	0	Negative	N/A
Blastocerus dichotomus	246B3	Negative	0	0	Negative	N/A
Blastocerus dichotomus	234B3	Negative	0	0	Negative	N/A
Blastocerus dichotomus	284B3	Positive	1	0.356	Negative	N/A
Blastocerus dichotomus	139B3	Negative	0	0	Negative	N/A
Blastocerus dichotomus	236B3	Negative	0	0	Negative	N/A
Blastocerus dichotomus	264B3	Negative	0	0	Negative	N/A
Blastocerus dichotomus	67B2	Negative	0	0	Negative	N/A
Desmodus rotundus	202MO	Negative	0	0	Negative	N/A
Cerdocyon thous	3L	Negative	0	0	Negative	N/A
Leopardus pardalis	7L	Negative	0	0	Negative	N/A
Cerdocyon thous	10L	Negative	0	0	Negative	N/A
Cerdocyon thous	13L	Negative	0	0	Negative	N/A
Cerdocyon thous	15L	Negative	0	0	Negative	N/A

Leopardus pardalis	16L	Negative	0	0	Negative	N/A
Cerdocyon thous	17L	Negative	0	0	Negative	N/A
Cerdocyon thous	19L	Negative	0	0	Negative	N/A
Leopardus pardalis	23L	Positive	1	0.356	Negative	N/A
Cerdocyon thous	24L	Negative	0	0	Negative	N/A
Cerdocyon thous	26L	Negative	0	0	Negative	N/A
Puma concolor	06P	Negative	0	0	Negative	N/A
Puma concolor	08P	Negative	0	0	Negative	N/A
Puma concolor	18P	Negative	0	0	Negative	N/A
Myrmecophaga tridactyla	97T	Negative	0	0	Negative	N/A
Blastocerus dichotomus	21B	Negative	0	0	Negative	N/A
Blastocerus dichotomus	23B	Negative	0	0	Negative	N/A
Blastocerus dichotomus	25B	Negative	0	0	Negative	N/A
Blastocerus dichotomus	27B	Negative	0	0	Negative	N/A
Blastocerus dichotomus	30B	Negative	0	0	Negative	N/A
Blastocerus dichotomus	31B	Negative	0	0	Negative	N/A
Blastocerus dichotomus	32B	Positive	1	0.362	Negative	N/A
Blastocerus dichotomus	33B	Negative	0	0	Negative	N/A
Blastocerus dichotomus	34B	Negative	0	0	Negative	N/A
Blastocerus dichotomus	36B	Negative	0	0	Negative	N/A
Blastocerus dichotomus	42B	Negative	0	0	Negative	N/A
Blastocerus dichotomus	44B	Negative	0	0	Negative	N/A
Blastocerus dichotomus	53B	Positive	1	0.356	Negative	N/A
Blastocerus dichotomus	54B	Negative	0	0	Negative	N/A
Blastocerus dichotomus	57B	Negative	0	0	Negative	N/A
Blastocerus dichotomus	59B	Negative	0	0	Negative	N/A
Blastocerus dichotomus	60B	Negative	0	0	Negative	N/A
Blastocerus dichotomus	66B	Negative	0	0	Negative	N/A
Blastocerus dichotomus	89B	Negative	0	0	Negative	N/A
Blastocerus dichotomus	90B	Negative	0	0	Negative	N/A
Blastocerus dichotomus	99B	Negative	0	0	Negative	N/A
Blastocerus dichotomus	105B	Positive	2	0.712	Negative	N/A
Blastocerus dichotomus	111B	Negative	0	0	Negative	N/A
Blastocerus dichotomus	113B	Negative	0	0	Negative	N/A
Blastocerus dichotomus	117B	Negative	0	0	Negative	N/A
Blastocerus dichotomus	123B	Negative	0	0	Negative	N/A
Blastocerus dichotomus	124B	Positive	1	0.361	Negative	N/A
Blastocerus dichotomus	126B	Negative	0	0	Negative	N/A
Blastocerus dichotomus	127B	Negative	0	0	Negative	N/A
Blastocerus dichotomus	135B	Negative	0	0	Negative	N/A
Blastocerus dichotomus	160B	Negative	0	0	Negative	N/A
Blastocerus dichotomus	229B	Negative	0	0	Negative	N/A
Blastocerus dichotomus	230B	Negative	0	0	Negative	N/A
Blastocerus dichotomus	235B	Negative	0	0	Negative	N/A
Blastocerus dichotomus	238B	Positive	2	0.713	Negative	N/A
Blastocerus dichotomus	241B	Negative	0	0	Negative	N/A
Blastocerus dichotomus	265B	Negative	0	0	Negative	N/A
Tapirus terrestris	3A	Negative	0	0	Negative	N/A
Tapirus terrestris	5A	Negative	0	0	Negative	N/A
Tapirus terrestris	6A	Negative	0	0	Negative	N/A
Tapirus terrestris	21A	Negative	0	0	Negative	N/A
Tapirus terrestris	95A	Negative	0	0	Negative	N/A
Tapirus terrestris	106A	Positive	1		Negative	N/A
Tapirus terrestris	104A	Negative	0	0	Negative	N/A

Tapirus terrestris	107A	Negative	0	0	Negative	N/A
Myrmecophaga tridactyla	163T	Negative	0	0	Negative	N/A
Myrmecophaga tridactyla	172T	Negative	0	0	Negative	N/A
Myrmecophaga tridactyla	202T	Negative	0	0	Negative	N/A
Myrmecophaga tridactyla	178T	Negative	0	0	Negative	N/A
Euphractus sexcinctus	100T	Negative	0	0	Negative	N/A
Euphractus sexcinctus	35T	Negative	0	0	Negative	N/A
Euphractus sexcinctus	25T	Negative	0	0	Negative	N/A
Tamandua tetradactyla	101T	Negative	0	0	Negative	N/A
Tamandua tetradactyla	133T	Negative	0	0	Negative	N/A
Tamandua tetradactyla	115T	Negative	0	0	Negative	N/A
Tamandua tetradactyla	57T	Negative	0	0	Negative	N/A
Tamandua tetradactyla	73T	Negative	0	0	Negative	N/A
Priodontes maximus	135T	Negative	0	0	Negative	N/A
Priodontes maximus	145T	Negative	0	0	Negative	N/A
Euphractus sexcinctus	150T	Positive	1	0.360	Negative	N/A
Priodontes maximus	154T	Positive	1	0.357	Negative	N/A
Dasypus novemcinctus	41T	Positive	1	0.357	Negative	N/A
Dasypus novemcinctus	42T	Negative	0	0	Negative	N/A
Dasypus novemcinctus	88T	Positive	1	0.356	Negative	N/A
Oecomys mamorae	58R	Negative	0	0	Negative	N/A
Oecomys mamorae	18R	Negative	0	0	Negative	N/A
Oecomys mamorae	48R	Negative	0	0	Negative	N/A
Oecomys mamorae	84R	Negative	0	0	Negative	N/A
Thrichomys fosteri	7R	Negative	0	0	Negative	N/A
Clyomis laticeps	42R	Positive	1	0.356	Negative	N/A
Thrichomys fosteri	43R	Negative	0	0	Negative	N/A
Thrichomys fosteri	13R	Negative	0	0	Negative	N/A
Thrichomys fosteri	60R	Positive	1	0.358	Negative	N/A
Leptotila verreauxi	107C	Negative	0	0	Negative	N/A
Icterus cayanensis	72C	Negative	0	0	Negative	N/A
Lepidocolaptes angustirostris	59C	Positive	1	0.357	Negative	N/A
Leptotila verreauxi	78C	Negative	0	0	Negative	N/A
Ramphocelus carbo	90C	Negative	0	0	Negative	N/A
Turdus leocomelas	85C	Negative	0	0	Negative	N/A
Fumarius rufus	119C	Negative	0	0	Negative	N/A
Saltator coerulescens	120C	Positive	1	0.356	Negative	N/A
Furnarius leucopus	117C	Negative	0	0	Negative	N/A
Cyanocorax chryrops	124C	Negative	0	0	Negative	N/A
Desmodus rotundus	162MO	Negative	0	0	Negative	N/A
Desmodus rotundus	180MO	Positive	1	0.356	Negative	N/A
Desmodus rotundus	158MO	Negative	0	0	Negative	N/A
Desmodus rotundus	223MO	Negative	0	0	Negative	N/A
Desmodus rotundus	200MO	Negative	0	0	Negative	N/A
Desmodus rotundus	224MO	Negative	0	0	Negative	N/A
Desmodus rotundus	222MO	Negative	0	0	Negative	N/A
Desmodus rotundus	201MO	Positive	3	1.070	Negative	N/A

Table S3: Results obtained for each test carried out for piroplasmids.

		dPCR			qPCR		nPCR
**Animal species**	**ID**	**Result**	**Positive Partitions**	**Conc (copies/µL)**	**Result**	**CQ**	**Result**
Subulo gouazoubira	182M	Positive	20	8.262	Negative	N/A	Positive
Mazama jucunda	215M	Positive	22	8.331	Negative	N/A	Positive
Mazama rufa	270M	Positive	13	4.934	Negative	N/A	Positive
Blastocerus dichotomus	63B	Positive	476	186	Negative	N/A	Positive
Blastocerus dichotomus	75B	Positive	36	13.68	Negative	N/A	Positive
Blastocerus dichotomus	120B2	Positive	121	46.02	Positive	28.51	Positive
Blastocerus dichotomus	121B2	Positive	55	19.97	Positive	29.25	Positive
Blastocerus dichotomus	222B3	Positive	125	47.65	Negative	N/A	Positive
Blastocerus dichotomus	226B3	Positive	12	4.290	Negative	N/A	Positive
Desmodus rotundus	91MO	Positive	8	2.849	Negative	N/A	Positive
Desmodus rotundus	8MO	Positive	2	0.750	Negative	N/A	Positive
Cerdocyon thous	2L	Positive	16	6.065	Positive	31.55	Positive
Leopardus pardalis	8L	Positive	3333	1830.1	Positive	25.52	Positive
Leopardus pardalis	22L	Positive	162	63.38	Negative	N/A	Positive
Cerdocyon thous	25L	Positive	4	1.560	Positive	33.27	Positive
Myrmecophaga tridactyla	59T	Positive	1	0.392	Negative	N/A	Positive
Myrmecophaga tridactyla	62T	Negative	0	0	Negative	N/A	Positive
Tamandua tetradactyla	71T	Positive	5	1.951	Positive	34.61	Positive
Dasypus novemcinctus	16T	Positive	112	44.69	Positive	29.11	Positive
Priodontes maximus	153T	Positive	1	0.357	Positive	34.71	Positive
Priodontes maximus	159T	Positive	4	1.562	Negative	N/A	Positive
Priodontes maximus	160T	Positive	2	1.453	Negative	N/A	Positive
Ozotocerus bezoarticus	06O	Positive	2	0.770	Negative	N/A	Positive
Ozotocerus bezoarticus	07O	Positive	18	6.974	Negative	N/A	Positive
Ozotocerus bezoarticus	08O	Positive	1	0.395	Negative	N/A	Positive
Ozotocerus bezoarticus	09O	Positive	7	2.733	Negative	N/A	Positive
Ozotocerus bezoarticus	10O	Positive	4	1.584	Negative	N/A	Positive
Subulo gouazoubira	154M	Positive	1	0.403	Negative	N/A	Positive
Subulo gouazoubira	155M	Positive	2	0.801	Negative	N/A	Positive
Subulo gouazoubira	156M	Negative	0	0.399	Negative	N/A	Positive
Subulo gouazoubira	180M	Positive	3	1.075	Negative	N/A	Positive
Desmodus rotundus	13MO	Positive	5	1.813	Negative	N/A	Positive
Desmodus rotundus	24MO	Positive	8	3.125	Positive	33.05	Positive
Desmodus rotundus	25MO	Positive	72	27.45	Negative	N/A	Positive
Desmodus rotundus	33MO	Positive	75	28.54	Negative	N/A	Positive
Desmodus rotundus	34MO	Positive	12	4.680	Negative	N/A	Positive
Desmodus rotundus	40MO	Positive	58	22.92	Negative	N/A	Positive
Desmodus rotundus	50MO	Positive	35	13.89	Negative	N/A	Positive
Desmodus rotundus	58MO	Positive	1	0.396	Negative	N/A	Positive
Desmodus rotundus	65MO	Positive	17	6.781	Negative	N/A	Positive
Desmodus rotundus	68MO	Positive	56	22.74	Positive	29.49	Positive

Cerdocyon thous	1L	Positive	6	2.407	Negative	N/A	Positive
Leopardus pardalis	4L	Positive	363	286.7	Positive	27.1	Positive
Leopardus pardalis	5L	Positive	2150	987.5	Positive	25.55	Positive
Leopardus pardalis	11L	Positive	1255	486.7	Positive	26.3	Positive
Leopardus pardalis	14L	Positive	1751	769.6	Positive	27.51	Positive
Leopardus pardalis	20L	Positive	1135	477.7	Positive	26.42	Positive
Leopardus pardalis	21L	Positive	3489	1784.1	Positive	25.12	Positive
Panthera onca	2P	Positive	133	53.97	Negative	N/A	Positive
Panthera onca	3P	Positive	147	60.26	Negative	N/A	Positive
Panthera onca	4P	Positive	966	413.8	Negative	N/A	Positive
Panthera onca	5P	Positive	64	26.29	Negative	N/A	Positive
Panthera onca	7P	Positive	387	160.9	Negative	N/A	Positive
Panthera onca	9P	Positive	1018	435.7	Negative	N/A	Positive
Panthera onca	11P	Positive	44	17.68	Negative	N/A	Positive
Panthera onca	12P	Positive	1049	443.1	Negative	N/A	Positive
Panthera onca	13P	Positive	63	24.22	Positive	34.49	Positive
Panthera onca	14P	Positive	911	372.3	Negative	N/A	Positive
Myrmecophaga tridactyla	51T	Negative	0	0	Positive	22.49	Positive
Myrmecophaga tridactyla	64T	Negative	0	0	Negative	N/A	Positive
Tamandua tetradactyla	66T	Negative	0	0	Negative	N/A	Positive
Myrmecophaga tridactyla	69T	Negative	0	0	Negative	N/A	Positive
Tamandua tetradactyla	72T	Negative	0	0	Negative	N/A	Positive
Myrmecophaga tridactyla	01T	Positive	7	2.837	Positive	35.54	Positive
Myrmecophaga tridactyla	03T	Positive	4	1.606	Positive	34.89	Positive
Myrmecophaga tridactyla	05T	Negative	0	0	Positive	36.84	Positive
Myrmecophaga tridactyla	06T	Positive	1	0.398	Negative	N/A	Positive
Tamandua tetradactyla	12T	Negative	0	0	Negative	N/A	Positive
Pseudoseisura unirufa	18C	Positive	3	1.071	Positive	35.51	Positive
Saltator coerulescens	27C	Positive	14	5.331	Positive	33.51	Positive
Turdus leocomelas	97C	Positive	80	32.00	Negative	N/A	Positive
Cercomacra melanaria	98C	Positive	115	45.37	Negative	N/A	Positive
Ramphocelus carbo	404C	Negative	0	0	Negative	N/A	Positive
Basileuterus flaveolus	405C	Positive	2	0.795	Negative	N/A	Positive
Saltator coerulescens	452C	Negative	0	0	Negative	N/A	Positive
Leptotila verreauxi	453C	Positive	1	0.401	Negative	N/A	Positive
Tapirus terrestris	1A	Positive	37	14.63	Negative	N/A	Positive
Tapirus terrestris	2A	Positive	345	145.8	Positive	33.56	Positive
Tapirus terrestris	10A	Positive	47	16.80	Negative	N/A	Positive
Tapirus terrestris	11A	Positive	21	8.231	Negative	N/A	Positive
Tapirus terrestris	63A	Positive	258	100.9	Negative	N/A	Positive
Tapirus terrestris	64A	Positive	8	3.047	Negative	N/A	Positive
Tapirus terrestris	75A	Positive	13	6.844	Negative	N/A	Positive

Tapirus terrestris	82A	Positive	1	0.381	Negative	N/A	Positive
Tapirus terrestris	90A	Positive	41	15.55	Negative	N/A	Positive
Tapirus terrestris	110A	Positive	15	5.707	Positive	35.59	Positive
Tapirus terrestris	112A	Positive	72	27.70	Positive	34.84	Positive
Tapirus terrestris	114A	Positive	12	4.595	Negative	N/A	Positive
Tapirus terrestris	118A	Positive	22	8.341	Negative	N/A	Positive
Tapirus terrestris	119A	Positive	15	5.659	Negative	N/A	Positive
Desmodus rotundus	176MO	Positive	7	2.585	Negative	N/A	Positive
Desmodus rotundus	182MO	Positive	12	4.615	Negative	N/A	Positive
Desmodus rotundus	214MO	Negative	0	0	Negative	N/A	Positive
Desmodus rotundus	215MO	Positive	75	28.58	Positive	29.49	Positive
Desmodus rotundus	216MO	Positive	24	8.578	Negative	N/A	Positive
Desmodus rotundus	218MO	Positive	16	6.065	Positive	34.69	Positive
Desmodus rotundus	219MO	Positive	3	1.154	Negative	N/A	Positive
Desmodus rotundus	220MO	Positive	6	2.261	Positive	36.06	Positive
Desmodus rotundus	221MO	Positive	3	1.129	Negative	N/A	Positive
Desmodus rotundus	231MO	Positive	38	14.37	Positive	35.33	Positive
Desmodus rotundus	205MO	Positive	9	3.358	Positive	34.29	Positive
Desmodus rotundus	19MO	Positive	32	11.95	Negative	N/A	Positive
Desmodus rotundus	27MO	Positive	10	3.776	Positive	38.36	Positive
Desmodus rotundus	35MO	Positive	22	8.345	Positive	35.38	Positive
Desmodus rotundus	76MO	Positive	10	3.866	Positive	35.2	Positive
Desmodus rotundus	82MO	Positive	8	3.115	Negative	N/A	Positive
Desmodus rotundus	115MO	Positive	1	0.392	Negative	N/A	Positive
Desmodus rotundus	90MO	Positive	1	0.390	Negative	N/A	Positive
Desmodus rotundus	170MO	Positive	3	1.168	Negative	N/A	Positive
Desmodus rotundus	179MO	Positive	32	12.66	Positive	35.19	Positive
Desmodus rotundus	207MO	Positive	2	0.713	Negative	N/A	Positive
Desmodus rotundus	63MO	Positive	812	484.3	Positive	35.31	Positive
Desmodus rotundus	70MO	Positive	5	1.781	Positive	36.06	Positive
Desmodus rotundus	88MO	Positive	3	1.129	Negative	N/A	Positive
Desmodus rotundus	92MO	Negative	0	0	Negative	N/A	Positive
Leopardus pardalis	9L	Positive	2302	1156.5	Positive	25.87	Positive
Panthera onca	15P	Positive	1826	1295.7	Positive	27.74	Positive
Panthera onca	16P	Positive	38	14.81	Negative	N/A	Positive
Panthera onca	17P	Positive	1583	818.6	Negative	N/A	Positive
Panthera onca	19P	Positive	1351	1265.0	Positive	28.17	Positive
Panthera onca	20P	Positive	1167	567.9	Negative	N/A	Positive
Panthera onca	21P	Positive	50	19.99	Positive	30.21	Positive
Panthera onca	22P	Positive	51	19.93	Positive	30.26	Positive
Panthera onca	23P	Positive	58	22.84	Positive	29.87	Positive
Panthera onca	24P	Positive	67	26.20	Positive	29.41	Positive
Panthera onca	25P	Positive	38	14.41	Positive	30.17	Positive
Myrmecophaga tridactyla	17T	Positive	1	0.378	Positive	41.27	Positive
Euphractus sexcinctus	32T	Positive	7	2.724	Positive	35	Positive

Myrmecophaga tridactyla	50T	Negative	0	0	Negative	N/A	Positive
Dasypus novemcinctus	76T	Negative	0	0	Positive	39.78	Positive
Dasypus novemcinctus	79T	Negative	0	0	Positive	38.36	Positive
Myrmecophaga tridactyla	81T	Positive	3	1.192	Negative	N/A	Positive
Dasypus novemcinctus	149T	Positive	1	0.404	Negative	N/A	Positive
Tapirus terrestris	122A	Positive	9	3.616	Negative	N/A	Positive
Tapirus terrestris	124A	Positive	7	2.787	Negative	N/A	Positive
Subulo gouazoubira	196M	Positive	14	5.489	Negative	N/A	Positive
Mazama jucunda	204M	Positive	16	6.242	Negative	N/A	Positive
Mazama jucunda	208M	Positive	7	2.671	Negative	N/A	Positive
Subulo gouazoubira	209M	Positive	2	0.766	Negative	N/A	Positive
Mazama jucunda	210M	Positive	7	2.729	Negative	N/A	Positive
Mazama rufa	267M	Negative	0	0	Negative	N/A	Positive
Mazama rufa	268M	Positive	6	2.365	Negative	N/A	Positive
Subulo gouazoubira	298M	Positive	3	1.216	Negative	N/A	Positive
Subulo gouazoubira	299M	Positive	22	8.842	Negative	N/A	Positive
Subulo gouazoubira	300M	Negative	0	0	Positive	17.37	Positive
Subulo gouazoubira	301M	Positive	11	4.462	Negative	N/A	Positive
Subulo gouazoubira	302M	Positive	3	1.201	Negative	N/A	Positive
Subulo gouazoubira	303M	Negative	0	0	Negative	N/A	Positive
Subulo gouazoubira	307M	Positive	6	2.362	Negative	N/A	Positive
Subulo gouazoubira	313M	Positive	3	1.077	Negative	N/A	Positive
Subulo gouazoubira	314M	Positive	4	1.541	Negative	N/A	Positive
Subulo gouazoubira	315M	Positive	9	3.480	Negative	N/A	Positive
Subulo gouazoubira	316M	Positive	1	0.395	Negative	N/A	Positive
Subulo gouazoubira	317M	Positive	4	1.610	Positive	43.57	Positive
Subulo gouazoubira	318M	Positive	2	0.713	Negative	N/A	Positive
Subulo gouazoubira	319M	Positive	5	2.007	Negative	N/A	Positive
Subulo gouazoubira	320M	Positive	27	10.94	Negative	N/A	Positive
Blastocerus dichotomus	35B	Positive	3	1.201	Negative	N/A	Positive
Blastocerus dichotomus	71B	Negative	0	0	Negative	N/A	Positive
Blastocerus dichotomus	78B	Positive	4	1.588	Negative	N/A	Positive
Blastocerus dichotomus	79B	Positive	3	1.067	Negative	N/A	Positive
Blastocerus dichotomus	94B	Positive	2	0.718	Negative	N/A	Positive
Blastocerus dichotomus	104B	Positive	1	0.379	Negative	N/A	Positive
Blastocerus dichotomus	24B	Positive	8	3.122	Negative	N/A	Positive
Blastocerus dichotomus	25B	Positive	231	91.70	Negative	N/A	Positive
Blastocerus dichotomus	26B	Positive	221	87.58	Positive	28.99	Positive
Blastocerus dichotomus	28B	Positive	67	26.71	Negative	N/A	Positive
Blastocerus dichotomus	29B	Positive	40	16.02	Positive	34.11	Positive
Blastocerus dichotomus	37B	Positive	3	1.200	Positive	31.82	Positive
Blastocerus dichotomus	38B	Positive	274	116.1	Positive	29.08	Positive
Blastocerus dichotomus	40B	Positive	16	6.245	Positive	18.9	Positive
Blastocerus dichotomus	41B	Positive	3498	2273.2	Positive	26.96	Positive
Blastocerus dichotomus	43B	Positive	17	6.056	Negative	N/A	Positive

Blastocerus dichotomus	45B	Positive	240	86.94	Positive	28.43	Positive
Blastocerus dichotomus	46B	Positive	4	1.484	Negative	N/A	Positive
Blastocerus dichotomus	48B	Positive	109	41.10	Positive	28.91	Positive
Blastocerus dichotomus	49B	Positive	740	412.2	Negative	N/A	Positive
Blastocerus dichotomus	51B	Positive	3	1.132	Negative	N/A	Positive
Blastocerus dichotomus	52B	Positive	172	67.51	Positive	28.51	Positive
Blastocerus dichotomus	55B	Positive	3	1.150	Negative	N/A	Positive
Blastocerus dichotomus	64B	Positive	8260	2.496	Negative	N/A	Positive
Blastocerus dichotomus	68B	Positive	2	0.756	Positive	35.76	Positive
Blastocerus dichotomus	76B	Positive	29	10.94	Negative	N/A	Positive
Desmodus rotundus	36MO	Positive	54	19.47	Negative	N/A	Positive
Desmodus rotundus	43MO	Positive	1	0.366	Negative	N/A	Positive
Desmodus rotundus	44MO	Positive	23	8.221	Negative	N/A	Positive
Desmodus rotundus	47MO	Positive	57	20.41	Positive	35.87	Positive
Desmodus rotundus	54MO	Positive	3	1.084	Negative	N/A	Positive
Blastocerus dichotomus	77B	Positive	4	1.441	Negative	N/A	Positive
Blastocerus dichotomus	78B	Positive	1082	414.9	Negative	N/A	Positive
Blastocerus dichotomus	80B	Positive	275	101.6	Positive	31.19	Positive
Blastocerus dichotomus	81B	Positive	10	3.561	Negative	N/A	Positive
Blastocerus dichotomus	83B	Positive	32	11.69	Negative	N/A	Positive
Blastocerus dichotomus	84B	Positive	9	3.256	Negative	N/A	Positive
Blastocerus dichotomus	86B	Positive	47	17.20	Negative	N/A	Positive
Blastocerus dichotomus	87B	Positive	7	2.492	Negative	N/A	Positive
Blastocerus dichotomus	88B	Positive	1396	546.4	Positive	38.14	Positive
Blastocerus dichotomus	92B	Positive	46	16.46	Negative	N/A	Positive
Blastocerus dichotomus	93B	Positive	74	26.77	Negative	N/A	Positive
Blastocerus dichotomus	98B	Positive	7	3.168	Negative	N/A	Positive
Blastocerus dichotomus	100B	Positive	103	36.94	Positive	35.15	Positive
Blastocerus dichotomus	102B	Positive	91	32.51	Negative	N/A	Positive
Blastocerus dichotomus	103B	Positive	115	41.31	Positive	32.24	Positive
Blastocerus dichotomus	106B	Positive	9	3.204	Negative	N/A	Positive
Blastocerus dichotomus	107B	Positive	33	11.78	Negative	N/A	Positive
Blastocerus dichotomus	108B	Positive	39	13.92	Negative	N/A	Positive
Blastocerus dichotomus	110B	Positive	26	9.284	Negative	N/A	Positive
Blastocerus dichotomus	112B	Positive	1131	433.5	Positive	38.81	Positive
Blastocerus dichotomus	128B	Positive	257	92.84	Positive	29.66	Positive
Blastocerus dichotomus	130B	Positive	16	5.718	Positive	38.8	Positive
Blastocerus dichotomus	131B	Positive	60	21.44	Positive	35.72	Positive
Blastocerus dichotomus	134B	Positive	21	8.231	Negative	N/A	Positive
Blastocerus dichotomus	136B	Positive	19	6.767	Negative	N/A	Positive
Blastocerus dichotomus	138B	Positive	8	2.854	Negative	N/A	Positive
Blastocerus dichotomus	25B1	Positive	45	16.08	Positive	32.82	Positive
Blastocerus dichotomus	50B1	Positive	247	89.43	Negative	N/A	Positive
Blastocerus dichotomus	58B1	Positive	1	0.356	Negative	N/A	Positive
Blastocerus dichotomus	65B1	Positive	6	2.139	Negative	N/A	Positive
Blastocerus dichotomus	74B1	Positive	16	5.727	Negative	N/A	Positive

Blastocerus dichotomus	82B1	Positive	9	3.221	Negative	N/A	Positive
Blastocerus dichotomus	95B1	Positive	23	8.220	Negative	N/A	Positive
Blastocerus dichotomus	129B1	Positive	168	60.57	Negative	N/A	Positive
Blastocerus dichotomus	101B1	Positive	9	3.206	Negative	N/A	Positive
Blastocerus dichotomus	32B2	Positive	48	17.19	Positive	35.32	Positive
Blastocerus dichotomus	72B2	Positive	153	55.00	Negative	N/A	Positive
Blastocerus dichotomus	85B2	Positive	20	7.145	Negative	N/A	Positive
Blastocerus dichotomus	97B2	Positive	3	1.068	Negative	N/A	Positive
Blastocerus dichotomus	109B2	Positive	13	4.636	Negative	N/A	Positive
Blastocerus dichotomus	113B2	Positive	83	29.66	Negative	N/A	Positive
Blastocerus dichotomus	114B2	Positive	217	78.28	Positive	29	Positive
Blastocerus dichotomus	115B2	Positive	1226	479.4	Positive	27.2	Positive
Blastocerus dichotomus	116B2	Positive	159	57.44	Positive	29.74	Positive
Blastocerus dichotomus	117B2	Positive	52	18.60	Negative	N/A	Positive
Blastocerus dichotomus	118B2	Positive	35	12.62	Negative	N/A	Positive
Blastocerus dichotomus	122B2	Positive	84	30.05	Negative	N/A	Positive
Blastocerus dichotomus	201B3	Positive	5	1.782	Negative	N/A	Positive
Blastocerus dichotomus	205B3	Positive	1	0.357	Negative	N/A	Positive
Blastocerus dichotomus	209B3	Positive	65	23.22	Positive	41.89	Positive
Blastocerus dichotomus	207B3	Positive	80	28.60	Positive	36.49	Positive
Blastocerus dichotomus	208B3	Positive	90	32.37	Negative	N/A	Positive
Blastocerus dichotomus	206B3	Positive	12	4.303	Negative	N/A	Positive
Blastocerus dichotomus	212B3	Positive	12	4.283	Negative	N/A	Positive
Blastocerus dichotomus	213B3	Positive	42	15.29	Negative	N/A	Positive
Blastocerus dichotomus	208MO	Negative	0	0	Negative	N/A	Negative
Blastocerus dichotomus	217B3	Positive	22	7.878	Negative	N/A	Positive
Blastocerus dichotomus	218B3	Positive	11	3.943	Negative	N/A	Positive
Blastocerus dichotomus	227B3	Positive	51	18.18	Negative	N/A	Positive
Blastocerus dichotomus	228B3	Negative	0	0	Negative	N/A	Positive
Blastocerus dichotomus	231B3	Positive	13	4.637	Negative	N/A	Positive
Blastocerus dichotomus	237B3	Negative	0	0	Negative	N/A	Positive
Blastocerus dichotomus	240B3	Positive	7	2.511	Negative	N/A	Positive
Blastocerus dichotomus	245B3	Positive	22	7.867	Negative	N/A	Positive
Blastocerus dichotomus	246B3	Positive	116	41.67	Negative	N/A	Positive
Blastocerus dichotomus	234B3	Negative	0	0	Negative	N/A	Positive
Blastocerus dichotomus	284B3	Positive	3	1.068	Negative	N/A	Positive
Blastocerus dichotomus	139B3	Positive	11	3.930	Negative	N/A	Positive
Blastocerus dichotomus	236B3	Positive	4	1.423	Negative	N/A	Positive
Blastocerus dichotomus	264B3	Positive	7	2.495	Negative	N/A	Positive
Blastocerus dichotomus	67B2	Positive	1	0.356	Negative	N/A	Positive
Desmodus rotundus	202MO	Positive	1	0.357	Positive	36.13	Negative
Cerdocyon thous	3L	Positive	3	1.069	Negative	N/A	Negative
Leopardus pardalis	7L	Positive	4	1.432	Negative	N/A	Negative
Cerdocyon thous	10L	Positive	3	1.071	Positive	37.06	Negative
Cerdocyon thous	13L	Positive	4	1.423	Positive	35.39	Negative
Cerdocyon thous	15L	Positive	4	1.427	Positive	36.25	Negative

Leopardus pardalis	16L	Positive	6	2.136	Positive	37.74	Negative
Cerdocyon thous	17L	Positive	2	0.713	Positive	36.13	Negative
Cerdocyon thous	19L	Positive	1	0.357	Positive	37.07	Negative
Leopardus pardalis	23L	Positive	5	1.782	Positive	38.62	Negative
Cerdocyon thous	24L	Positive	4	1.425	Positive	38.39	Negative
Cerdocyon thous	26L	Positive	1	0.357	Positive	37.2	Negative
Puma concolor	06P	Positive	52	18.62	Negative	N/A	Negative
Puma concolor	08P	Positive	75	26.93	Negative	N/A	Negative
Puma concolor	18P	Positive	56	20.05	Positive	35.24	Negative
Myrmecophaga tridactyla	97T	Negative	0	0	Negative	N/A	Negative
Blastocerus dichotomus	21B	Negative	0	0	Negative	N/A	Negative
Blastocerus dichotomus	23B	Negative	0	0	Positive	40.62	Negative
Blastocerus dichotomus	25B	Positive	554	206.2	Positive	29.38	Negative
Blastocerus dichotomus	27B	Negative	0	0	Negative	N/A	Negative
Blastocerus dichotomus	30B	Positive	653	243.9	Positive	29.11	Negative
Blastocerus dichotomus	31B	Positive	297	108.4	Positive	29.58	Negative
Blastocerus dichotomus	32B	Positive	2	0.723	Negative	N/A	Negative
Blastocerus dichotomus	33B	Positive	214	83.69	Positive	29.75	Negative
Blastocerus dichotomus	34B	Positive	106	38.18	Positive	31.41	Negative
Blastocerus dichotomus	36B	Negative	0	0	Negative	N/A	Negative
Blastocerus dichotomus	42B	Negative	0	0	Negative	N/A	Negative
Blastocerus dichotomus	44B	Positive	138	49.77	Positive	39.16	Negative
Blastocerus dichotomus	53B	Positive	325	118.1	Positive	30.57	Negative
Blastocerus dichotomus	54B	Negative	0	0	Negative	N/A	Negative
Blastocerus dichotomus	57B	Positive	5	1.782	Negative	N/A	Negative
Blastocerus dichotomus	59B	Negative	0	0	Negative	N/A	Negative
Blastocerus dichotomus	60B	Positive	1	0.357	Negative	N/A	Negative
Blastocerus dichotomus	66B	Positive	83	29.74	Positive	32.4	Negative
Blastocerus dichotomus	89B	Positive	4678	2921.6	Positive	32.94	Negative
Blastocerus dichotomus	90B	Positive	520	191.1	Positive	30.46	Negative
Blastocerus dichotomus	99B	Negative	0	0	Negative	N/A	Negative
Blastocerus dichotomus	105B	Positive	9	3.204	Negative	N/A	Negative
Blastocerus dichotomus	111B	Positive	2105	869.4	Positive	29.37	Negative
Blastocerus dichotomus	113B	Positive	2076	856.1	Positive	29.36	Negative
Blastocerus dichotomus	117B	Positive	83	29.74	Positive	32.51	Negative
Blastocerus dichotomus	123B	Positive	148	53.29	Positive	32.61	Negative
Blastocerus dichotomus	124B	Positive	1	0.361	Negative	N/A	Negative
Blastocerus dichotomus	126B	Negative	0	0	Negative	N/A	Negative
Blastocerus dichotomus	127B	Positive	2	0.713	Positive	39.71	Negative
Blastocerus dichotomus	135B	Negative	0	0	Positive	40.53	Negative
Blastocerus dichotomus	160B	Negative	0	0	Negative	N/A	Negative
Blastocerus dichotomus	229B	Positive	163	58.70	Positive	29.13	Negative
Blastocerus dichotomus	230B	Positive	1133	435.1	Positive	32	Negative
Blastocerus dichotomus	235B	Positive	631	234.6	Positive	29.58	Negative
Blastocerus dichotomus	238B	Positive	323	117.5	Positive	30.58	Negative
Blastocerus dichotomus	241B	Positive	1754	713.4	Positive	27.43	Negative

Blastocerus dichotomus	265B	Positive	1	0.357	Negative	N/A	Negative
Tapirus terrestris	3A	Positive	15	5.346	Negative	N/A	Negative
Tapirus terrestris	5A	Positive	386	153.8	Positive	37.33	Negative
Tapirus terrestris	6A	Positive	104	37.27	Negative	N/A	Negative
Tapirus terrestris	21A	Positive	14	4.989	Negative	N/A	Negative
Tapirus terrestris	95A	Positive	61	21.79	Negative	N/A	Negative
Tapirus terrestris	106A	Positive	5	1.782	Negative	N/A	Negative
Tapirus terrestris	104A	Positive	11	3.920	Negative	N/A	Negative
Tapirus terrestris	107A	Positive	6	2.138	Negative	N/A	Negative
Myrmecophaga tridactyla	163T	Positive	12	4.276	Positive	36.1	Negative
Myrmecophaga tridactyla	172T	Positive	8	2.848	Negative	N/A	Negative
Myrmecophaga tridactyla	202T	Positive	9	3.204	Negative	N/A	Negative
Myrmecophaga tridactyla	178T	Positive	18	6.415	Negative	N/A	Negative
Euphractus sexcinctus	100T	Positive	1	0.357	Positive	37.01	Negative
Euphractus sexcinctus	35T	Negative	0	0	Negative	N/A	Negative
Euphractus sexcinctus	25T	Negative	0	0	Negative	N/A	Negative
Tamandua tetradactyla	101T	Negative	0	0	Negative	N/A	Negative
Tamandua tetradactyla	133T	Positive	1	0.356	Positive	43.63	Negative
Tamandua tetradactyla	115T	Negative	0	0	Negative	N/A	Negative
Tamandua tetradactyla	57T	Negative	0	0	Negative	N/A	Negative
Tamandua tetradactyla	73T	Negative	0	0	Negative	N/A	Negative
Priodontes maximus	135T	Negative	0	0	Negative	N/A	Negative
Priodontes maximus	145T	Positive	1	0.356	Negative	N/A	Negative
Euphractus sexcinctus	150T	Positive	1	0.360	Negative	N/A	Negative
Priodontes maximus	154T	Positive	1	0.357	Negative	N/A	Negative
Dasypus novemcinctus	41T	Negative	0	0	Negative	N/A	Negative
Dasypus novemcinctus	42T	Negative	0	0	Negative	N/A	Negative
Dasypus novemcinctus	88T	Negative	0	0	Negative	N/A	Negative
Oecomys mamorae	58R	Positive	108	38.81	Positive	35.42	Negative
Oecomys mamorae	18R	Positive	56	20.05	Positive	34.86	Negative
Oecomys mamorae	48R	Positive	32	11.45	Positive	35.49	Negative
Oecomys mamorae	84R	Positive	68	24.35	Negative	N/A	Negative
Thrichomys fosteri	7R	Positive	91	32.58	Negative	N/A	Negative
Clyomis laticeps	42R	Positive	124	44.53	Negative	N/A	Negative
Thrichomys fosteri	43R	Positive	45	16.09	Positive	38.03	Negative
Thrichomys fosteri	13R	Positive	44	15.90	Negative	N/A	Negative
Thrichomys fosteri	60R	Positive	14	5.013	Negative	N/A	Negative
Leptotila verreauxi	107C	Positive	1	0.360	Positive	40.74	Negative
Icterus cayanensis	72C	Positive	1	0.357	Positive	37.42	Negative
Lepidocolaptes angustirostris	59C	Positive	1	0.357	Negative	N/A	Negative
Leptotila verreauxi	78C	Negative	0	0	Negative	N/A	Negative
Ramphocelus carbo	90C	Negative	0	0	Negative	N/A	Negative
Turdus leocomelas	85C	Negative	0	0	Negative	N/A	Negative

Fumarius rufus	119C	Positive	2	0.714	Negative	N/A	Negative
Saltator coerulescens	120C	Positive	1	0.356	Positive	38.9	Negative
Furnarius leucopus	117C	Positive	2	0.713	Negative	N/A	Negative
Cyanocorax chryrops	124C	Positive	1	0.356	Positive	40.71	Negative
Desmodus rotundus	162MO	Negative	0	0	Negative	N/A	Negative
Desmodus rotundus	180MO	Negative	0	0	Negative	N/A	Negative
Desmodus rotundus	158MO	Positive	2	0.712	Negative	N/A	Negative
Desmodus rotundus	223MO	Positive	1	0.357	Negative	N/A	Negative
Desmodus rotundus	200MO	Positive	3	1.070	Positive	40.9	Negative
Desmodus rotundus	224MO	Positive	1	0.357	Negative	N/A	Negative
Desmodus rotundus	222MO	Positive	4	1.429	Negative	N/A	Negative
Desmodus rotundus	201MO	Positive	3	1.070	Negative	N/A	Negative

Supplementary Figure S1

Venn diagrams comparing the positivity between different techniques for the detection of *Bartonella* spp. (A), *Borrelia* spp. (B), and piroplasmids (C), considering samples as positive in dPCR when presenting one or more, two or more, or three or more positive partitions in the assays performed.

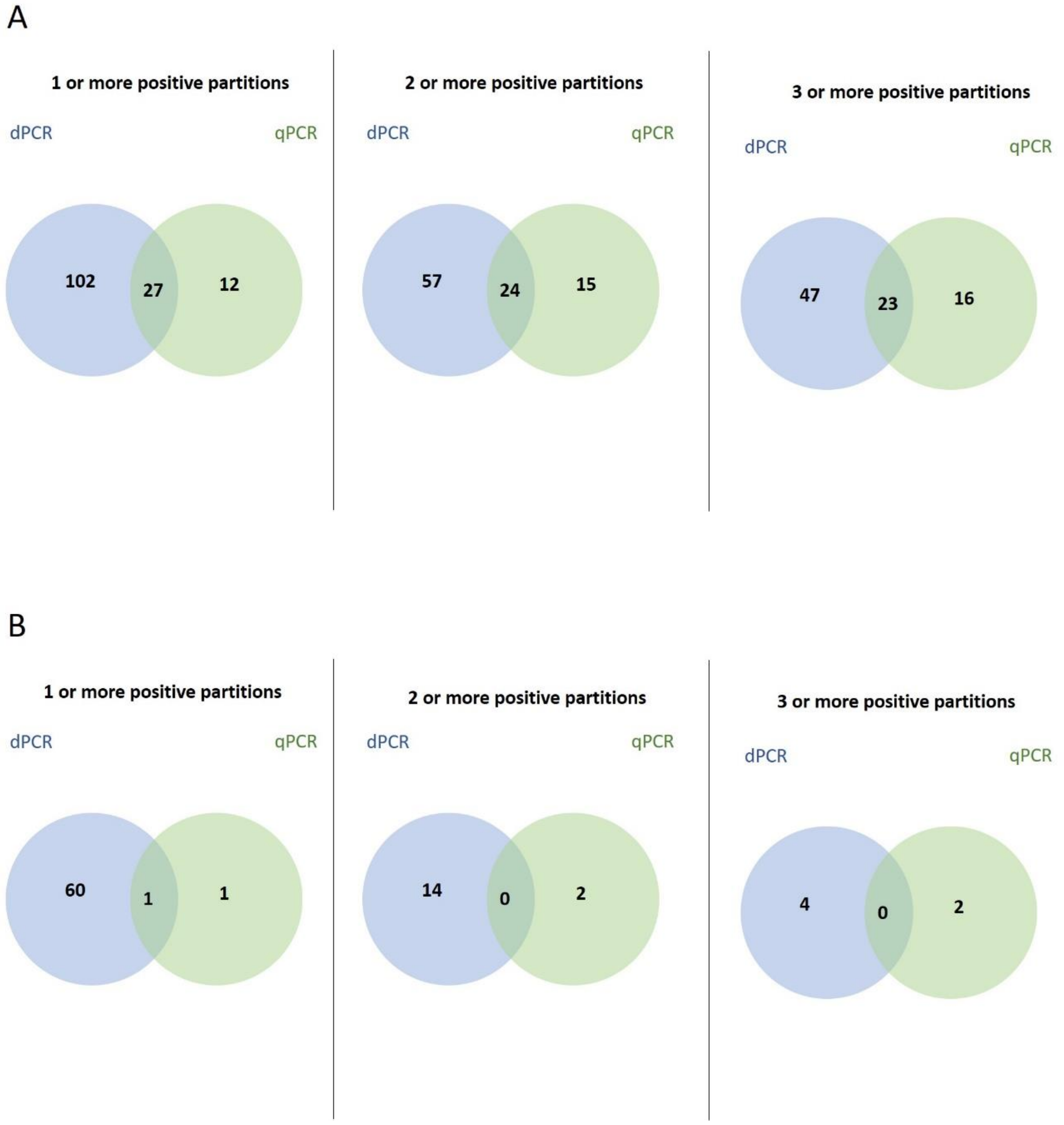

C

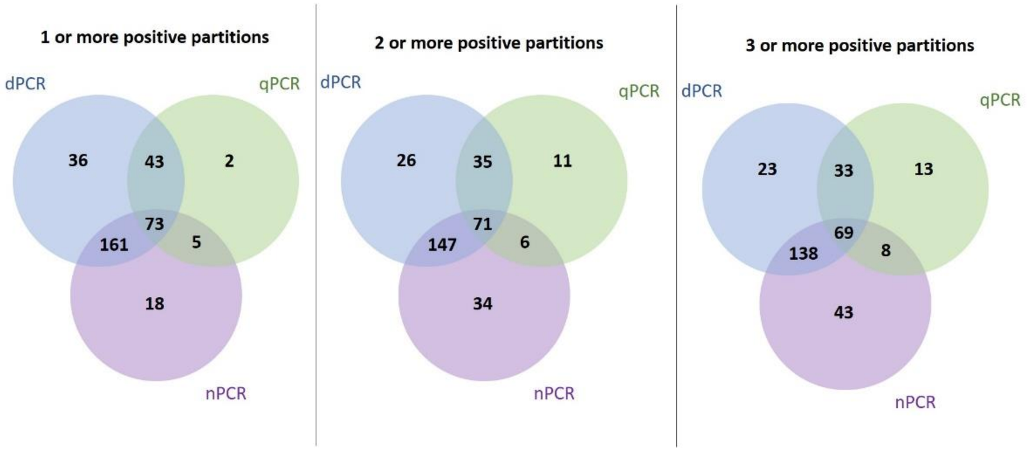
1 or more positive partitions
dPCR
qPCR
nPCR
36
43
2
73
161
5
18
2 or more positive partitions
dPCR
qPCR
nPCR
26
35
11
71
147
6
34
3 or more positive partitions
dPCR
qPCR
nPCR
23
33
13
69
138
8
43
